# Genome analyses of amphotericin B-susceptible and -resistant strains of *Leishmania* (*Mundinia*) *martiniquensis* reveal variations potentially related to amphotericin B resistance

**DOI:** 10.1016/j.crpvbd.2025.100255

**Published:** 2025-03-18

**Authors:** Narissara Jariyapan, Sivamoke Dissook, Pitiporn Noisagul, Patcharawadee Thongkumkoon, Chonlada Mano, Romteera Kittichaiworakul, Anuluck Junkum, Adisak Tantiworawit, Pascale Pescher, Gerald F. Späth, Hatim Almutairi, Padet Siriyasatien

**Affiliations:** a Center of Excellence in Vector Biology and Vector-Borne Disease, Department of Parasitology, Faculty of Medicine, Chulalongkorn University, Bangkok, 10330, Thailand; b Department of Biochemistry, Faculty of Medicine, Chiang Mai University, Chiang Mai, 50200, Thailand; c Center of Multidisciplinary Technology for Advanced Medicine, Faculty of Medicine, Chiang Mai University, Chiang Mai, 50200, Thailand; d Parasitology and Entomology Research Cluster (PERC), Department of Parasitology, Faculty of Medicine, Chiang Mai University, Chiang Mai, 50200, Thailand; e Department of Internal Medicine, Faculty of Medicine, Chiang Mai University, Chiang Mai, 50200, Thailand; f Institut Pasteur, INSERM U1201, Université Paris Cité, Unité de Parasitologie Moléculaire et Signalisation, 75015, Paris, France; g Department of Genome and Data, National Livestock and Fisheries Development Program, Ministry of Environment, Water and Agriculture, Riyadh, Saudi Arabia

**Keywords:** *Leishmania*, *Leishmania martiniquensis*, Amphotericin B, Resistance, Ergosterol, Genome, Thailand

## Abstract

Amphotericin B deoxycholate (AmpB) is used for the treatment of leishmaniasis caused by *Leishmania* (*Mundinia*) *martiniquensis* in Thailand, and relapse cases have been documented. To date, genomic analysis of drug-resistant *L*. *martiniquensis* strains is limited. In this study, comparative genome analyses were performed with an experimentally selected AmpB-resistant *L*. *martiniquensis* (AmpBRP2i) and two cryopreserved *L*. *martiniquensis* parasite strains isolated from a patient showing differences in response to AmpB treatment, LSCM1-WT (susceptible) and LSCM1-6 (resistant). Applying the GIP genome analyses package, we identified aneuploidy and gene copy number variations in all three samples, none of which correlated with AmpB resistance. In contrast, single nucleotide variant (SNV) analyses revealed an SNV in AmpB-resistant strains introduced a premature stop codon into a putative sterol C-24 reductase gene (*C24R*) (*LSCM1_02556*) involved in the ergosterol biosynthetic pathway in *Leishmania*. As *Leishmania* AmpB resistance has previously been linked to mutations in other genes of the ergosterol biosynthesis pathway in different species of *Leishmania* parasites, these results suggest that *C24R* may serve as an additional marker of AmpB resistance in *Leishmania*. We further identified two missense SNVs in AmpB-resistant strains in a putative ‘ABC transporter-like/ABC transporter family’ gene (*LSCM1_01856*) that could be involved in drug efflux. These initial findings pave the way for future research with a larger number of isolates to confirm the genomic signature we associate here with AmpB resistance.

## Highlights

• Genome analysis of AmpB-susceptible and AmpB-resistant *L. martiniquensis* strains revealed variations.
• Gene copy number variations and aneuploidy were not correlated with AmpB resistance.
• One nonsense SNV in a *C24R* (*LSCM1_02556*) was found in the resistant strains.
• Two missense SNVs were found in a “ABC transporter-like/ABC transporter family” gene (*LSCM1_01856*) of the resistant strains.

## Graphical abstract

**Figure:**
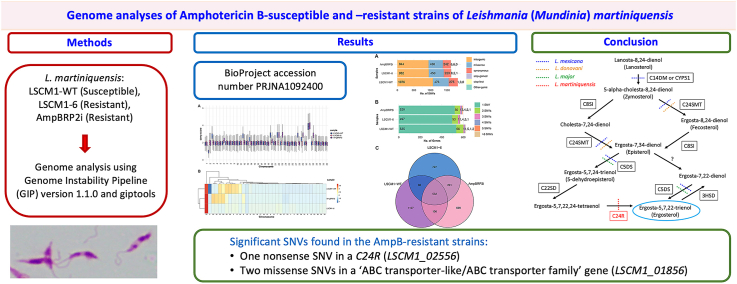

## 1 Introduction

Leishmaniasis is a vector-borne neglected tropical disease caused by at least 21 species of protozoan parasites of the genus *Leishmania* (WHO, 2023). *Leishmania* spp. are obligate intracellular parasites that reside in cells of the reticuloendothelial system of vertebrate hosts. Depending on the species, *Leishmania* infection results in three major forms of the disease, including cutaneous leishmaniasis (CL), mucocutaneous leishmaniasis (MCL), and visceral leishmaniasis (VL). Female sand flies of the genera *Phlebotomus* and *Lutzomyia* serve as natural vectors. Recent evidence of natural infection in an undescribed species of day-feeding midges in subgenus *Forcipomyia* (*Lasiohelea*) Kieffer with *Leishmania* (*Mundinia*) *macropodum* (Dougall et al., 2011) and in *Culicoides peregrinus* (Diptera: Ceratopogonidae) with *Leishmania* (*Mundinia*) *martiniquensis* (Kaewmee et al., 2023) supports that midges are potential vectors of *Leishmania*.

Amphotericin B deoxycholate (AmpB) is the first-line treatment for leishmaniasis in many countries. The general mode of action of AmpB is binding to ergosterol present in the cell membranes of *Leishmania* parasites leading to the formation of small membranous pores that cause instability of the parasite membranes, increase membrane permeability, induce ion leakage, and finally, cell death. AmpB also produces oxidative damage to the cells with the formation of reactive oxygen species (ROS) and subsequently increased membrane permeability (Kumar et al., 2022; Wijnant et al., 2022). AmpB has been introduced for the treatment of visceral leishmaniasis caused by *Leishmania donovani* in antimonial-non-responsive regions of Bihar, India, and at least two AmpB-resistant *L. donovani* strains isolated from leishmaniasis patients in India have been reported (Srivastava et al., 2011; Purkait et al., 2012; Goyal et al., 2020). Recent studies on *in vitro* selected AmpB-resistant strains of *Leishmania mexicana* and analyses on mechanisms of the resistance have revealed mutations in different genes in the ergosterol biosynthetic pathway in *L. mexicana*, i.e. genes encoding sterol C-14 demethylase (C14DM), sterol C-5 desaturase (C5DS), and sterol C-24-methyltransferase (C24SMT) (Mwenechanya et al., 2017; Pountain et al., 2019; Alpizar-Sosa et al., 2022). AmpB resistance in one strain of *L. donovani* was associated with a deletion of the gene *LinJ.36.2510* coding for C24SMT (Rastrojo et al., 2018). Additionally, Ning et al. (2020) have demonstrated that lathosterol oxidase (sterol C-5 desaturase) deletion confers resistance to AmpB in *Leishmania major*.

The appearance of leishmaniasis in Thailand has raised an important concern that there is already a much higher incidence of infection than reflected by the number of clinically and parasitologically confirmed cases. For example, a prevalence of 25.1% and 7.1% is indicated in HIV patients in southern and northern Thailand, respectively, most of whom are asymptomatic (Manomat et al., 2017; Sriwongpan et al., 2021). The most prominent etiological agent of leishmaniasis in Thailand is *L.* *martiniquensis*. Patients with no underlying immunodeficiency infected with *L. martiniquensis* show a range of clinical presentations, most frequently presenting VL. However, when accompanied by HIV infection, VL and/or disseminated cutaneous leishmaniasis (DCL) and/or MCL have been reported (Chiewchanvit et al., 2015; Srivarasat et al., 2022). Previously, we had successfully isolated *L. martiniquensis* parasites from a VL patient in the northern part of Thailand. Additionally, experimentally derived *L. martiniquensis* AmpB-resistant parasites (AmpBRP2i isolate) were obtained by drug selection *in vitro* (Mano et al., 2023a). Comparative analyses of fitness and *in vitro* susceptibility to AmpB and miltefosine (MIL) of the wild type and the *L. martiniquensis* AmpB-resistant parasites have revealed increased fitness and resistance to MIL (Mano et al., 2023a, 2023b). These results raise important questions regarding the role of genetic variations in the drug resistance of the parasites.

Comparative analyses of whole genome sequences using computational tools allow for the exploration of *Leishmania* genomes in depth with high resolution and accuracy providing data on genetic heterogeneity, such as changes in chromosome/gene copy numbers (gene dosage alterations) and single nucleotide polymorphisms (Downing et al., 2011; Rogers et al., 2011; Jeddi et al., 2014; Dumetz et al., 2017; Iantorno et al., 2017; Prieto Barja et al., 2017; Bussotti et al., 2021; Ghosh et al., 2022). Genome-wide analyses also reveal variations correlating with geographical and/or environmental adaptation (Imamura et al., 2016; Bussotti et al., 2018, 2020; Zackay et al., 2018; Sarraf et al., 2021) and drug resistance (Leprohon et al., 2015; Dumetz et al., 2018, Ghosh et al., 2022). To date, whole genome sequences of several strains of *L. martiniquensis* have been deposited in the NCBI GenBank database, including *L. martiniquensis* (MAR LEM2494) (https://www.ncbi.nlm.nih.gov/datasets/genome/GCA_000409445.2/), *L. martiniquensis* (LSCM1 isolate, LV760 strain) (Almutairi et al., 2021), *L. martiniquensis* (CU1 isolate) (Anuntakarun et al., 2022), and *L. martiniquensis* (PCM3 isolate) (Anuntasomboon et al., 2022). Additionally, three related genomes of *L*. *martiniquensis*, *L*. *enriettii*, and *L*. *macropodum* have also been reported (Butenko et al., 2019). Nevertheless, genomic analyses specifically focused on drug resistance in *L. martiniquensis* strains remain scarce.

Therefore, the objective of this study was to analyze and compare whole genomes of *L. martiniquensis* clinical isolates from a leishmaniasis patient (CM1) in the northern part of Thailand, that have differences in the susceptibility to AmpB treatment, LSCM1-WT (susceptible) and LSCM1-6 (resistant), and the experimentally derived *L. martiniquensis* AmpB-resistant (AmpBRP2i) strain. These genome analyses provided initial information regarding genetic variations related to AmpB resistance for further studies on the AmpB-resistance mechanisms in *L. martiniquensis*.

## 2 Materials and methods

### 2.1 *Leishmania* strains and culture

Three samples of *L. martiniquensis* were used in this study. Two of them were cryopreserved parasite strains isolated from a leishmaniasis patient from the northern part of Thailand including LSCM1-WT (original isolate called LSCM1) and LSCM1-6. Another sample was AmpBRP2i, the laboratory-generated strain derived from gradual AmpB pressure applied to the LSCM1-WT strain (Mano et al., 2023a) (Table 1). All isolates were thawed from cryopreservation and cultured in Schneider’s Insect Medium (SIM) (Sigma-Aldrich, St. Louis, MO, USA), pH 6.8, supplemented with 10% heat-inactivated fetal bovine serum (FBS) (Life Technologies-Gibco, Grand Island, NY, USA) and 25 μg/ml gentamicin sulfate (Sigma-Aldrich, St. Louis, MO, USA) at 26 °C. Parasites were sub-passaged in fresh medium for 7 passages and then used for genomic DNA extraction.

**Table 1:** Background information of three parasite samples (strains) used in this study.

Source	Parasite sample (strain)	First treatment	Relapse/treatment failure and treatment received	Reference
Parasite strains that are isolated from the CM1 patient of visceral leishmaniasis caused by *L. martiniquensis* from the northern part of Thailand without HIV co-infection	LSCM1-WT	Intravenous AmpB (1 mg/kg/d) for 3 weeks	–	Pothirat et al. (2014)
	LSCM1-6	–	About 1 year after the first treatment; over 5 years, 6 episodes of relapse occurred, and the patient had been given at least 6 courses of the treatment with AmpB	Pothirat et al. (2014); Mano et al. (2023a)
AmpB-resistant strain experimentally selected from the LSCM1-WT isolate	AmpBRP2i	–	–	Mano et al. (2023a)

### 2.2 Drug and promastigote susceptibility assay

Amphotericin B deoxycholate (Life Technologies-Gibco, Grand Island, NY, USA) as a 250 μg/ml solution solubilized in sodium deoxycholate was used. The stock solution was stored at −20 °C and used within 12 months. *In vitro* drug susceptibility on promastigotes was evaluated using alamarBlue® assay (Thermo Fisher Scientific Inc., Waltham, MA, USA). Fifty microliters of the logarithmic phase promastigotes (2 × 10^6^ cells/ml) were added in each well of 96-well flat-bottomed tissue culture plates and incubated at 26 °C for 1 h. Two-fold dilutions of AmpB from 27.703 to 0.002 μM were prepared in Eppendorf tubes, and 50 μl of each dilution were then added to the pre-plated parasites. Thereafter, the plate was incubated for 48 h. Then, 10 μl of alamarBlue® reagent was added to each well and incubated further for 24 h. The concentration of resorufin in the parasite-drug mixture was measured by spectrophotometry at 570 and 600 nm. The optical density in the absence of drugs was set as 100% control. Drug susceptibility was determined by calculating the half-maximal inhibitory concentration (IC_50_) values from the nonlinear concentration-response curves using GraphPad Prism version 9.1 software (Graphpad Software Inc., San Diego, CA, USA). The resistance index (IC_50_ value of AmpBRP2i or LSCM1-6 divided by IC_50_ value of LSCM1-WT) was also calculated. Results were expressed as the mean ± standard deviation (SD) of three independent experiments in triplicates.

### 2.3 DNA extraction and whole genome sequencing (WGS)

The genomic DNA of the LSCM1-WT, LSCM1-6, and AmpBRP2i samples was extracted using a genomic DNA purification kit (Thermo Fisher Scientific Inc., Waltham, MA, USA) according to the manufacturer’s instructions. The quantity and quality of the DNA were measured using a spectrophotometer (Nanodrop®, Thermo Fisher Scientific, Wilmington, DE, USA). Approximately 1 μg of DNA was used for WGS. The sequencing (150 paired-end) was conducted using Novaseq 6000 sequencing at NovogeneAIT Genomics Singapore Pte Ltd (Singapore).

### 2.4 Genome analysis of individual strain and comparative genomic analyses

Genome Instability Pipeline (GIP) version 1.1.0 and giptools (Späth and Bussotti, 2022) were applied to analyze and compare genomes of the LSCM1-WT, LSCM1-6, and AmpBRP2i samples. The reference genome of *L. martiniquensis* isolate LSCM1, strain LV760 (GenBank assembly accession: GCA_017916325.1) (Almutairi et al., 2021) was used in this study based on criteria such as completeness, assembly quality, and representativeness for comparative analyses focused specifically on amphotericin B resistance. For raw WGS of each isolate, FastQC (version 0.12.1) (https://www.bioinformatics.babraham.ac.uk/projects/fastqc/) and Cutadapt (version 4.5) (Martin, 2011) were used to remove low-quality bases and adapter contaminations. BWA mem (version 0.7.17) (Li and Durbin, 2009) was employed for the alignment. Subsequently, Picard CollectAlignmentSummaryMetrics and Picard CollectInsertSizeMetrics (version 2.18.9) (http://broadinstitute.github.io/picard) were used to estimate sequencing and mapping statistics. The resulting alignment files were sorted, indexed, and reformatted using Samtools (version 1.8) (Danecek et al., 2021). To remove read duplicates, Picard MarkDuplicates (http://broadinstitute.github.io/picard) was applied. Genes for cluster analysis and SNV calling were selected based on a minimum read alignment of a mean mapping quality (MAPQ) score of 50. Additionally, gene function annotation (predicted protein function) for *L. martiniquensis* isolate LSCM1, strain LV760 was performed using EGG-NOG mapper v. 2.1.9) (Cantalapiedra et al., 2021). Unmapped reads and scaffold regions were excluded from the analysis. The paired-end Illumina reads of each isolate were aligned to the reference genome to compute the mean sequencing coverage of genomic bins (windows size 300 bp) and genes. Gene coverage scores were normalized by median chromosome coverage to highlight amplifications or depletions with respect to the chromosome copy number. Freebayes v. 0.9.2 (Garrison and Marth, 2012) was used for SNV calling and followed by snpEff v. 5.1 (Cingolani et al., 2012) for variant effect annotation. Outputs of the GIP were used as input for giptools for comparative genome analyses and visualization of chromosome copy number variants (chromosome CNVs), gene copy number variants (gene CNVs), and single nucleotide variants (SNVs). The gene ontology (GO) terms of each annotated protein were obtained from Interproscan (Jones et al., 2014) then searched for data available in InterPro Classification of protein families (Paysan-Lafosse et al., 2023). BLAST search was performed against the NCBI database (Johnson et al., 2008). Clustal Omega was used to identify and align sequences (Madeira et al., 2022).

## 3 Results

### 3.1 Promastigote susceptibility to AmpB and characteristics of each sample

The IC_50_ value of the LSCM1-WT, LSCM1-6, and AmpBRP2i samples was determined to confirm susceptibility to AmpB before using the samples for DNA extraction and whole genome sequencing. The IC_50_ value of the LSCM1-WT representing strain before treatment was 0.052 ± 0.01 μM. For the resistant LSCM1-6 isolate and the *in vitro* induced resistant strain AmpBRP2i, the IC_50_ values were 0.165 ± 0.01 and 0.204 ± 0.01 μM, respectively, and thus up to 4-fold higher than that of the LSCM1-WT sample (Table 2). The resistance indices for LSCM1-6 and AmpBRP2i were 3.17 and 3.92, respectively (Table 2).

**Table 2:** Characteristics of the three samples of *L. martiniquensis* and their promastigote susceptibility to AmpB.

Characteristics	Parasite sample		
	LSCM1-WT	LSCM1-6	AmpBRP2i
Exposure to AmpB	Before	After	After
AmpB-susceptible or -resistant (IC_50_ value, μM)	Susceptible (0.052 ± 0.01)^a^	Resistant (0.165 ± 0.01)	Resistant (0.204 ± 0.01)
Resistance index	–	3.17	3.92

a Mean ± standard deviation based on three independent replicates in triplicates.

### 3.2 Genomic analysis of *L. martiniquensis* samples

The genome of all *L. martiniquensis* samples consisted of 36 chromosomes. The estimated size of the genomes and the number of genes were based on the reference genome which are 32.4 Mbp and 7967 genes (Almutairi et al., 2021). The sequencing statistics for each *L. martiniquensis* sample are shown in Table 3. Genomic sequences were submitted to the NCBI genome database with the BioProject accession number PRJNA1092400.

**Table 3:** Sample reports of *L. martiniquensis* samples analyzed using GIP.

Sample reports	Parasite sample		
	LSCM1-WT	LSCM1-6	AmpBRP2i
Median of genomic bin intervals	33.253	37.207	30.800
Genome bins coverage: Significantly amplified regions/depleted regions	24/0	18/0	10/0
Genome coverage: Significantly amplified genes/depleted genes	19/0	22/4	24/0
Total SNVs	1831	1636	1628

### 3.3 Chromosome copy number variation

**Fig. 1:**
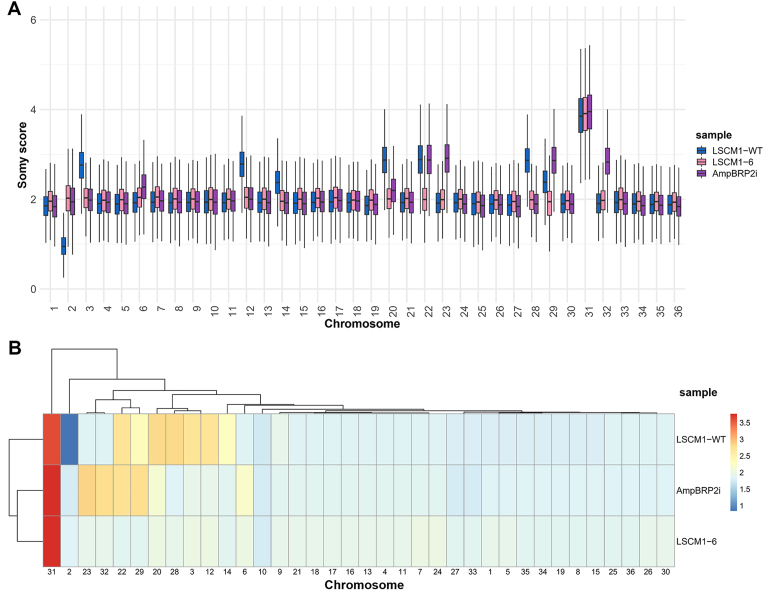
Aneuploidy in the LSCM1-WT, LSCM1-6, and AmpBRP2i samples. **A** Karyotype plot of somy scores for the 36 chromosomes in the three *Leishmania* samples. **B** Heatmap showing the estimated somy score for each chromosome and each sample (Supplementary Table S1). Color scale indicates somy scores. The left dendrogram identifies distinct clusters based on the similarity of aneuploidy profiles. The top dendrogram identifies the correlation between chromosomes.

The estimated somy scores of the 36 chromosomes for the three *L. martiniquensis* samples were diverse and showed a strain-specific distribution. Chromosome aneuploidy analysis reveals that chromosome 31 displayed a tetrasomic state in all *L. martiniquensis* samples (Supplementary Table S1). Trisomic states were found for chromosomes 3, 12, 20, 22, and 28 of the LSCM1-WT sample and chromosomes 22, 23, 29, and 32 of the AmpBRP2i sample, while all chromosomes except for chromosome 31 were disomic in the isolate LSCM1-6 resistant to AmpB. Conversely only LSCM1-WT and AmpBRP2i were diploid for the same 21 chromosomes (chromosomes 1, 4–11, 15–19, 21, 24–27, 30, and 33–36). Interestingly, only the LSCM1-WT presented a monosomic state for the chromosome 2 and a trisomic state for the chromosome 28 whilst both AmpB-resistant samples LSCM1-6 and AmpBRP2i were strictly disomic for both (Fig. 1A and B).

### 3.4 Gene copy number variation

Evaluation on the genetic variations among the three samples was performed by comparing the normalized sequencing coverage to detect gene CNV. The values of normalized sequencing coverage of each gene in each sample are shown in Supplementary Table S2. However, this analysis did not reveal any specific gene CNV in the “AmpB-resistant” group. Further analyses in SNVs were conducted to investigate other mechanisms related AmpB resistance of the parasite strains.

### 3.5 Single nucleotide variant analyses

**Fig. 2:**
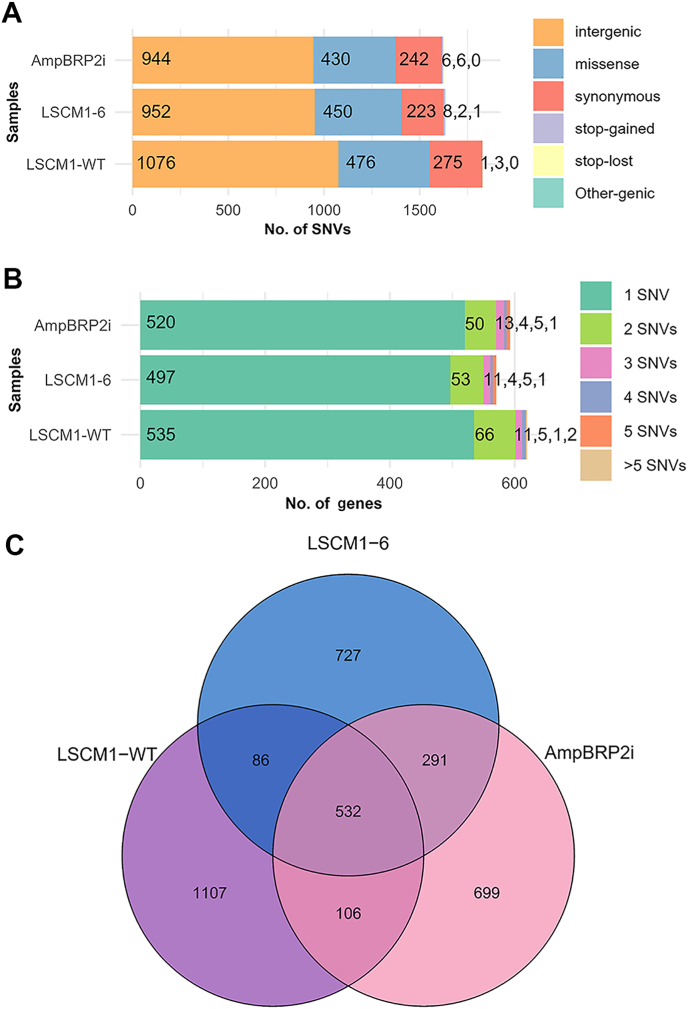
**A** The distribution of SNV types in the LSCM1-WT, LSCM1-6, and AmpBRP2i samples: intergenic (*orange*), missense (*blue*), synonymous (*red*), stop gained (*purple*), stop-loss (*yellow*), and other-genic (*green*). The number inside the columns indicates the number of SNVs in each type in each strain. **B** The distribution of the number of SNVs in each gene found in the six strains. Subcategories, 1SNV (*green*), 2SNVs (*light green*), 3SNVs (*pink*), 4SNVs (*gray*), 5SNVs (*orange*), and > 5SNVs (*brown*), are shown. The number inside the columns indicates the number of each SNV subcategory in each strain. **C** Venn diagram showing the number of unique and shared SNVs among the three studied strains.

The distribution of SNV types and the number of SNVs found in each gene of the three samples, and the number of unique and shared SNVs among the strains are shown in Fig. 2. In comparison to the reference genome, five types of SNVs were identified (Supplementary Table S3). The main SNV types found in the three samples were intergenic, missense, and synonymous variants. The number of stop-gained SNVs in the LSCM1-6 was greater than that in the AmpBRP2i and LSCM1-WT samples. The number of SNVs with stop-loss variants were 6, 3, and 2 in the AmpBRP2i, LSCM1-WT, and LSCM1-6, respectively (Fig. 2A). Additionally, the number of genes with one SNV was found dominantly in the three samples, i.e. 535, 520 and 497 genes in the LSCM1-WT, AmpBRP2i, and LSCM1-6 samples, respectively (Fig. 2B).

In this study, a total of 291 shared SNVs were identified in the “AmpB-resistant” group (Fig. 2C). Further analyses were focused on SNVs with allele frequency of 1 indicating a variation of a single nucleotide found in the whole genome (100% change) (Supplementary Table S4). Two high-impact SNVs were classified as a premature stop codon (stop-gained) and identified in both LSCM1-6 and AmpBPR2i samples (Table 4). The first SNV (CM030423.1_176446_G_T, n.396G>T; p.Tyr132∗) was found in the *LSCM1_03062* gene on chromosome 28 annotated for TPR_2 (a tetratricopeptide repeat 2 motif). Alignment of the hypothetical protein LSCM1_03062 with the corresponding protein in other *Leishmania* species revealed highly conserved regions (Supplementary Fig. S1). BLASTP results showed that the sequence of LSCM1_03062 was more similar to other *Leishmania* species of the subgenus *Mundinia* (90.49–91.47%) than the subgenus *Leishmania* and *Viannia* (85.29–86.99%) (Supplementary Fig. S2). The second one (CM030428.1_288118_G_A, n.183G>A; p.Trp61∗) was found in the *LSCM1_02556* gene on chromosome 33 annotated for *ERG4_ERG24* (ergosterol biosynthesis ERG4/ERG24 family) involved in the ergosterol biosynthetic pathway. Mutation or loss of genes involved in the ergosterol pathway causes altered susceptibility and leads to resistance to AmpB in *Leishmania* parasites (Ellis, 2002). BLASTP results and alignment analysis revealed that the hypothetical protein LSCM1_02556 was corresponding to a putative sterol C-24 reductase found in several *Leishmania* species, such as *L*. *infantum*, *L*. *mexicana*, *L*. *braziliensis*, *L*. *panamensis*, and *L. guyanensis* (Supplementary Figs. S3 and S4). Sequence identities of the LSCM1 reference strain compared to the other members in the subgenus *Mundinia*, *Leishmania*, and *Viannia* were 89.11–89.92%, 86.97%, and 82.40–82.60%, respectively (Supplementary Fig. S4).

**Table 4:** Specific SNVs with allele frequency > 0.2 of coding genes, motifs, or domains, and high or moderate impact in the “AmpB-resistant” group, LSCM1-6 and AmpBRP2i samples.

Variant type (Impact)	SNV	Gene ID/Chromosome	Change in coding sequence		Functional annotation; [GO terms: MF, BP, CC]
			Nucleotide	Amino acid	
Stop gained variant (High)	CM030423.1_176446_G_T	LSCM1_03062/28	396G>T	Tyr132∗	TPR_2; [MF - protein binding, Hsp90 protein binding]
	CM030428.1_288118_G_A	LSCM1_02556/33	183G>A	Trp61∗	ERG4_ERG24; [MF - oxidoreductase activity, acting on the CH-CH group of donors, NAD or NADP as acceptor, BP - sterol biosynthetic process, CC - membrane]
Missense variant (Moderate)	CM030403.1_390338_C_T	LSCM1_07222/8	3362G>A	Gly1121Glu	AAA_5; [MF - ATP hydrolysis activity, ATP binding]
	CM030403.1_395993_T_G	LSCM1_07223/8	1156A>C	Lys386Gln	DUF3586,Inhibitor_I29,Peptidase_C1; [MF - cysteine-type peptidase activity, cysteine-type endopeptidase activity, BP - proteolysis, proteolysis involved in protein catabolic process, CC - extracellular space, lysosome]
	CM030403.1_397069_C_G		80G>C	Gly27Ala	
	CM030403.1_417233_G_A	LSCM1_07227/8	313G>A	Gly105Arg	zf-RING_2; [MF - zinc ion binding, ubiquitin protein ligase activity]
	CM030403.1_417860_G_A		940G>A	Val314Ile	
	CM030411.1_97915_A_G	LSCM1_05662/16	7783A>G	Asn2595Asp	Ins145_P3_rec,Ion_trans,RIH_assoc,RYDR_ITPR; [MF - monoatomic ion channel activity, BP - calcium ion transmembrane transport, monoatomic ion transport, transmembrane transport, calcium ion transport, CC- membrane]
	CM030412.1_115208_T_C	LSCM1_05496/17	259T>C	Cys87Arg	M20_dimer,Peptidase_M20,Peptidase_M28,Peptidase_M42; [–]
	CM030412.1_121915_A_G	LSCM1_05499/17	2252A>G	Asp751Gly	Kinesin; [MF- microtubule motor activity, ATP binding, microtubule binding, ATP hydrolysis activity, BP - microtubule-based movement, CC - kinesin complex, microtubule]
	CM030412.1_657033_G_A	LSCM1_05631/17	1111G>A	Asp371Asn	Ferrochelatase; [MF - ferrochelatase activity, heme biosynthetic process]
	CM030416.1_347815_C_T	LSCM1_04684/21	1001C>T	Pro334Leu	DUF4042; [–]
	CM030419.1_753429_G_C	LSCM1_05162/24	196G>C	Glu66Gln	TFCD_C; [MF - GTPase activator activity, beta-tubulin binding, BP - microtubule cytoskeleton organization, protein folding, tubulin complex assembly, post-chaperonin tubulin folding pathway]
	CM030421.1_951732_G_A	LSCM1_04258/26	3308G>A	Arg1103Gln	Pkinase; [MF - protein kinase activity, ATP binding, BP - protein phosphorylation, mitotic spindle organization, regulation of cytokinesis, CC - spindle microtubule, spindle pole centrosome, chromosome passenger complex, spindle midzone]
	CM030422.1_942183_T_C	LSCM1_03570/27	1721T>C	Leu574Pro	WD40; [MF - protein binding]
	CM030422.1_949323_A_C	LSCM1_03573/27	512A>C	Glu171Ala	Thioredoxin; [BP - microtubule cytoskeleton organization, CC - cytoplasm]
	CM030426.1_459606_C_T	LSCM1_01415/31	34C>T	Pro12Ser	Cyt-b5; [–]
	CM030427.1_808052_T_G	LSCM1_01856/32	1833T>G	His611Gln	ABC_tran; [MF - ATP binding, ABC-type transporter activity, ATP hydrolysis activity, ATPase-coupled transmembrane transporter activity, BP - transmembrane transport, CC - membrane]
	CM030427.1_809533_G_A		352G>A	Gly118Arg	
	CM030428.1_1189531_G_A	LSCM1_02763/33	1733G>A	Gly578Glu	Kinesin; [MF -microtubule motor activity, ATP binding, ATP hydrolysis activity, microtubule binding, BP - microtubule-based movement, cytoskeleton-dependent intracellular transport, CC - kinesin complex, microtubule]
	CM030428.1_1226672_C_G	LSCM1_02773/33	454C>G	Pro152Ala	ThiF; [MF - ubiquitin-like modifier activating enzyme activity, NEDD8 activating enzyme activity, BP - protein modification by small protein conjugation, protein neddylation, CC - cytoplasm]

*Abbreviations*: MF, Molecular Function; BP, Biological Process; CC, Cellular Component; ∗, stop sign.

Additionally, 19 missense SNVs with a moderate impact were identified in 16 functionally annotated genes including the *LSCM1_07222* (AAA_5), *LSCM1_07223* (DUF3586,Inhibitor_I29,Peptidase_C1), *LSCM1_07227* (zf-RING_2), *LSCM1_05662* (Ins145_P3_rec,Ion_trans,RIH_assoc,RYDR_ITPR), *LSCM1_05496* (M20_dimer,Peptidase_M20,Peptidase_M28,Peptidase_M42), *LSCM1_05499* (Kinesin), *LSCM1_05631* (Ferrochelatase), *LSCM1_04684* (DUF4042), *LSCM1_05162* (Tubulin folding cofactor D C terminal, TFCD_C), *LSCM1_04258* (Pkinase), *LSCM1_03570* (WD40), *LSCM1_03573* (Thioredoxin), *LSCM1_01415* (Cyt-b5), *LSCM1_02763* (Kinesin), *LSCM1_02773/33* (ThiF), and *LSCM1_01856* (ABC transporter) (Table 4, Supplementary Table S4).

Of these genes, ABC transporters are considered to be proteins involved in the transport of many drugs and may be related to the resistance mechanism of *Leishmania* parasites; however, the exact effects leading to treatment failure remain controversial (Croft et al., 2006). LSCM1_01856 has previously been annotated as an ABC transporter-like protein in multiple *Leishmania* species. However, in this study, we identified two missense SNVs specifically associated with amphotericin B-resistant strains of *L. martiniquensis*, indicating that these variants might play a role in the drug efflux mechanism. Further functional studies are essential to determine whether these variants directly contribute to resistance (Supplementary Figs. S5 and S6).

## 4 Discussion

Mechanisms of drug resistance in *Leishmania* parasites are diverse and importantly often reflect the mechanism of action of the drug. At least two mechanisms involved in AmpB resistance in several *Leishmania* species include (i) reduction of ergosterol and/or changes in the composition of sterols in the cell membrane resulting in decreased AmpB-ergosterol binding in the plasma membrane (Mwenechanya et al., 2017), and (ii) activation of numerous enzymes involved in protection against ROS, such as tryparedoxin peroxidase (Suman et al., 2016), polyamine-trypanothione pathway (PTP) (Purkait et al., 2012), and the silent information regulator 2 (Sir2) protein (Purkait et al., 2015).

**Fig. 3:**
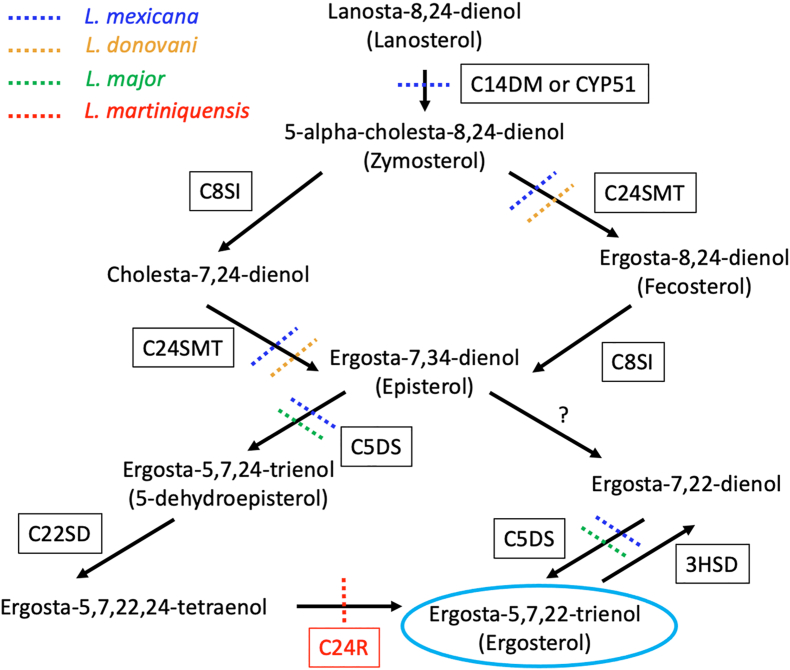
Summary of ergosterol biosynthesis pathway in *Leishmania* spp. adapted from Alpizar-Sosa et al. (2022) showing mutation observed in the gene(s) of *L. mexicana* (Mwenechanya et al., 2017; Pountain et al., 2019; Alpizar-Sosa et al., 2022), *L. donovani* (Rastrojo et al., 2018), *L. major* (Ning et al., 2020), and *L. martiniquensis* (this study) indicated with blue, orange, green, and red dotted lines, respectively.

In this study, the analyses of whole genome sequencing data from the three *L. martiniquensis* samples revealed that no correlation was observed with respect to karyotypic change or gene copy number variation and AmpB resistance. Interestingly, in both AmpB-resistant *L. martiniquensis* samples, a specific stop-gained SNV was found in the *LSCM1_02556* gene annotated for putative sterol C-24 reductase, an enzyme in the ergosterol biosynthetic pathway in *Leishmania* spp. This suggested that no functional protein was expressed from this gene in the *L. martiniquensis* AmpB-resistant parasites. Ergosterol, the major sterol in *Leishmania* spp. and yeasts, is required for plasma membrane organization. It is synthesized through the ergosterol biosynthetic pathway involving numerous enzymatic steps, i.e. C14DM, also known as lanosterol 14-α-demethylase (CYP51), C24SMT, C-8-sterol isomerase (C8SI), C5DS, sterol C-22 desaturase or cytochrome p450-like protein (C22SD), sterol C-24 reductase (C24R), and 3-beta hydroxysteroid dehydrogenase (3HSD) (Yao and Wilson, 2016; Mukherjee et al., 2019; Alpizar-Sosa et al., 2022). Here, to the best of our knowledge, for the first time, we link mutation at the gene annotated for C24R to AmpB-resistance in *Leishmania*. This enzyme acts at the final step of the ergosterol biosynthesis pathway by converting ergosta-5,7,22,24-tetraenol (ergostatetranol) to ergosta-5,7,22-trienol (ergosterol) (Fig. 3). The mutation observed in the AmpB-resistant *L. martiniquensis* strains likely abolishes C24R expression, likely affecting sterol metabolism and causing loss of AmpB binding to ergosterol.

So far, mutations of genes involved in the reduction of ergosterol have been identified in several species of AmpB-resistant *Leishmania*. Thus, C14DM or CYP51 leads to AmpB resistance in *L. mexicana* (Mwenechanya et al., 2017). In another study on AmpB-resistant *L. mexicana*, genomic changes (mutations) to one or the other of two genes encoding for sterol C5-desaturase (C5DS, *LmxM.23.1300*) and sterol C24-methyl transferase (C24SMT, *LmxM.36.2380* and *LmxM.36.2390*) have been identified with whole genome sequencing in 15 *in vitro* selected resistant cell lines (Alpizar-Sosa et al., 2022). The sterol intermediate accumulated in the resistant line(s) with the mutation of C5DS is ergosta-7,22-dienol, whereas cholesta-5,7,22-trienol is found in the resistant line(s) with the mutation of C24SMT. Lines with mutations in C5DS have shown both retained virulence and resistance to AmpB in mice. In contrast, for the lines with mutations in C24SMT, no lesion at the inoculation site (footpad) and no parasites were detected using histology, suggesting a fitness loss in the C24SMT mutant lines (Alpizar-Sosa et al., 2022). In the case of *L. donovani*, cholesta-5,7,24-trienol has been reported as a major sterol accumulated in the AmpB-resistant *L. donovani* parasites isolated from a VL patient in India, indicating changes to the C24SMT gene. Thus, the study indicates that the C24SMT-mutant *L. donovani* can persist in human infections (Purkait et al., 2012).

In our previous study, we demonstrated that the AmpB-resistant *L. martiniquensis* human isolate (LSCM1-6) and the *in vitro* derived AmpB-resistant strain (AmpBRP2i) can infect BALB/c mice and survive in the animals longer than the AmpB-susceptible strain (LSCM1-WT) (Mano et al., 2023a). We hypothesized that one of the abundant sterol intermediates accumulated could be ergosta-5,7,22,24-tetraenol and other intermediates could still be in the parasites. The accumulation of sterol intermediates could compensate for the damage to the parasite membrane permeability and, therefore, could explain the survival of the LSCM1-6 and AmpBRP2i strains in the mice. However, further studies on sterol profiling in *L. martiniquensis* are required to prove this hypothesis.

Another gene in this study that might be involved in a drug efflux pump was *LSCM1_01856* annotated for the ABC transporter-like/ABC transporter family, showing two missense SNVs. ATP-binding cassette (ABC) transporters are found in various species of pathogens, including *Leishmania*. The ABC proteins use ATP hydrolysis to exclude multiple compounds across the membranes and have a crucial role in the resistance to drugs by two mechanisms, i.e. overexpression and mutation of ABC-carrying genes. The ABC transporters play a significant role in the ability of parasites to resist multiple drugs by actively pumping them out of the cell; this is a key mechanism contributing to drug resistance in parasite infections (Coelho and Cotrim, 2018). In *Leishmania* spp., certain members of the ABC transporter family are considered multidrug resistance proteins (MRPs) and are associated with treatment failure (Croft et al., 2006; Boozhmehrani et al., 2022).

Overexpression of the *ABCB4* is often associated with increased resistance to drugs like AmpB in *Leishmania* parasites. Purkait et al. (2012) have shown that the expression of the MDR1 transporter, encoded by the *MDR1* (or *ABCB4*), is higher in AmpB-resistant *L. donovani*. A study on the expression of ABC transporter genes, i.e. *ABCC3*, *ABCC7*, *ABCI4*, *ABCB4*, and *ABCG2*, in *L. major* antimonial-treatment failure isolates has demonstrated that the ABC transporter genes in each isolate have a different gene expression pattern (Boozhmehrani et al., 2022). For example, *ABCB4* overexpresses as 1.04- to 44.63-fold in treatment failure isolates, whereas *ABCI4* and *ABCC7* expression patterns are the same in treatment failure isolates with overexpression in most isolates (6/8). Thus, the mRNA modification and expression of the *LSCM1_01856* in *L. martiniquensis* should be performed to analyze whether it involves resistance to AmpB of the parasite species or not.

In the present study, the *LSCM1_03062* encoding for TPR_2, a stop-gained SNV (nonsense variant), and 15 other functionally annotated genes harboring missense variants predicted to have moderate impacts on protein function were also identified (Table 4). Up to date, little information regarding these genes involved in drug resistance or treatment failure of *Leishmania* spp. is available. Therefore, further investigations to elucidate the specific biological functions of these genes or their associated protein domains and their potential roles in the important biological processes of *Leishmania* parasites are necessary.

Furthermore, studies on resistance to AmpB of *L*. *donovani* and *L*. *mexicana* have shown that during the process of selection of drug resistance lines, the miltefosine transporter gene (LdMT in *L*. *donovani* or LmxM.13.1530 in *L*. *mexicana*) is also mutated or deleted (Fernandez-Prada et al., 2016; Shaw et al., 2016; Pountain et al., 2019). The miltefosine transporter gene is a P-type ATPase gene that is involved in the translocation of MIL and glycerophospholipids across the plasma membrane of *Leishmania* parasites. Our previous study has found that the LSCM1-6 and AmpBRP2i strains are cross-resistant to MIL (Mano et al., 2023b). Thus, further studies are needed to elucidate genes that might be involved in cross-resistance with MIL of both *L*. *martiniquensis*-resistant strains.

Although random genomic variations can occur independently of drug pressure, the repeated association of specific SNVs with AmpB resistance strongly suggests a selection mechanism linked explicitly to drug exposure. Alternative explanations could be due to genomic plasticity, founder effects, or culture-driven selection independent of drug pressure, which might also enrich specific SNVs. Future studies with broader comparative genomic analyses and parallel drug-free cultures could help distinguish among these possibilities.

Indeed, spontaneous mutations across various genomic regions can occur frequently in *Leishmania* parasites due to environmental adaptation, such as during development in culture media, insects, and vertebrate hosts, reflecting their intrinsic genomic plasticity. In this study, some degree of genetic variation is expected between the LSCM1-WT and the LSCM1 reference genomes. Although both the LSCM1-WT strain studied and the LSCM1 reference genome originated from the same isolate (LSCM1), in our previous study, the LSCM1-WT strain was inoculated in BALB/c mice and recovered from the liver of the infected mice to avoid loss of parasite virulence before cultivation and cryopreservation for further use (Mano et al., 2023a). This protocol is commonly used in many laboratories. Thus, this might affect the genomic plasticity of the parasites.

Finally, our findings highlight the emergence of AmpB-resistant *L. martiniquensis* in Thailand. Understanding the genetic basis of AmpB resistance in *L. martiniquensis* has direct epidemiological and public health relevance. Specifically, the identification of genomic markers such as mutations in C24R and ABC transporter genes may enhance molecular surveillance strategies, enabling earlier detection of drug-resistant parasites. This knowledge can inform clinical practice by potentially guiding treatment choices, thus reducing therapeutic failures and relapse rates. Additionally, our findings highlight the necessity for routine genomic monitoring as part of integrated public health strategies, especially in endemic regions such as Thailand.

## 5 Conclusions

Our initial results reveal a stop-gained SNV (nonsense variant) identified in the *LSCM1_02556* gene annotated for *C24R*, indicating no functional protein derived from this gene. It is suggesting that the *LSCM1_*02556 might be potentially related to AmpB resistance in *L. martiniquensis*, which has been previously linked to mutations in other genes of the ergosterol biosynthesis pathway, such as *C14DM* encoding sterol C-14 demethylase, *C24SMT* encoding sterol C-24-methyltransferase, and *C5DS* encoding sterol C-5 desaturase, reported for different species of *Leishmania*. Another gene that might be involved in drug efflux membrane transporters was the *LSCM1_01856* gene with two missense SNVs annotated for the ABC transporter-like/ABC transporter family. However, before drawing any conclusions, further investigation with a larger number of isolates from patients should be performed to confirm the mutation gene associated with AmpB resistance.

## CRediT authorship contribution statement

**Narissara Jariyapan:** Conceptualization, Methodology, Investigation, Formal analysis, Visualization, Funding acquisition, Project administration, Resources, Writing – original draft, Writing – review & editing. **Sivamoke Dissook:** Methodology, Formal analysis, Investigation, Validation, Funding acquisition, Writing – review & editing. **Pitiporn Noisagul:** Methodology, Investigation, Formal analysis, Validation, Visualization, Data curation, Writing – review & editing. **Patcharawadee Thongkumkoon:** Methodology, Investigation, Formal analysis, Validation, Visualization, Data curation, Writing – review & editing. **Chonlada Mano:** Investigation, Visualization, Writing – review & editing. **Romteera Kittichaiworakul:** Investigation, Writing – review & editing. **Anuluck Junkum:** Resources, Writing – review & editing. **Adisak Tantiworawit:** Resources, Writing – review & editing. **Pascale Pescher:** Formal analysis, Writing – review & editing. **Gerald F. Späth:** Methodology, Formal analysis, Writing – review & editing. **Hatim Almutairi:** Validation, Writing – review & editing. **Padet Siriyasatien:** Funding acquisition, Resources, Writing – review & editing.

## Ethical approval

The parasites used in this laboratory experiment were acquired from the cryopreserved parasite repository and are not associated with any identifiable private information of patients, therefore, the informed consent is waived from the requirement. The protocol was conducted in compliance with the CIOMS International Ethical Guidelines for Health-related Research, and the protocol was submitted to and exempted by the Institution Review Board (IRB) of the Faculty of Medicine, Chulalongkorn University (COE No. 031/2022) before the study began.

## Data availability

Data supporting the conclusions of this article are included within the article and its supplementary files. Genomic sequences have been submitted to the NCBI genome database with the BioProject accession number PRJNA1092400.

## Funding

This study was supported by the French Ministry of Europe and Foreign Affairs (10.13039/501100003388MEAE), the French 10.13039/100020552Ministry of Higher Education, Research and Innovation (MESRI), the 10.13039/501100002385Ministry of Higher Education, Science, Research and Innovation of Thailand (10.13039/501100016204MHESI) - Franco-Thai Mobility Program/10.13039/100012453PHC SIAM 2021–2022 (Grant no. 10.13039/100012453PHC SIAM, 2021–2022), the 10.13039/501100010724Health Systems Research Institute (10.13039/501100010724HSRI), Thailand (Grant no.66-144), and Genomic Thailand, the 10.13039/501100010724Health Systems Research Institute (10.13039/501100010724HSRI), Thailand (Grant no. 67-118).

## Declaration of competing interests

The authors declare that they have no known competing financial interests or personal relationships that could have appeared to influence the work reported in this paper.
