# Supplementary material for: Genome analyses of amphotericin B-susceptible and -resistant strains of Leishmania (Mundinia) martiniquensis reveal variations potentially related to amphotericin B resistance
Source: Curr Res Parasitol Vector Borne Dis. 2025 Mar 18;7:100255. doi: 10.1016/j.crpvbd.2025.100255 (PMC11984574; doi:10.1016/j.crpvbd.2025.100255)
Supplement: Multimedia component 1 [file mmc1.pdf]

Fig. S1 Alignment of the protein sequences corresponding to the hypothetical protein LSCM1_03062 [*Leishmania martiniquensis*]. From top to bottom parasites listed are *L.* (*M.*) *martiniquensis*, *L.* (*M.*) *enriettii*, *L.* sp. *Namibia* (*L.* (*M.*) *procaviensis* n. sp.), *L.* sp. *Ghana* (*L.* (*M.*) *chancei* n. sp.), *L.* (*M.*) *orientalis*, *L.* (*V.*) *braziliensis*, *L.* (*V.*) *panamensis*, *L.* (*L.*) *mexicana*, *L.* (*L.*) *major*, *L.* (*L.*) *infantum*, *L.* (*L.*) *donovani*, *L.* (*L.*) *donovani.* Proteins sequences are from Blastp (https://blast.ncbi.nlm.nih.gov/Blast.cgi). Alignment was performed using Clustal Omega (https://www.ebi.ac.uk/jdispatcher/msa/clustalo). Note: The LSCM1_03062 sequence is similar to the TPR repeat family protein sequence of *L.* (*L.*) *donovani*.

```
CLUSTAL O(1.2.4) multiple sequence alignment


LSCM1_03062_L.mar                MQMPLDVPAATAAEEDTLISLCPEDDGDVPDMPFFAASNLEDRPIAPAAEDYPLGCEVEL      60
KAG5474951.1_L.enr               MQMFLD--VAAAAEENTLISLCPEDDGDVPDMPFFAASNLEDRPVAPAAEDYPLGCEVEL      58
KAG5498199.1_L.Nam               MQMFLD--VAAAAEENTLISLCPEDDGDVPDMPFFAASNLEDRPVAPAAEDYPLGCEVEL      58
KAG5499776.1_L.Gha               MQMFLD--VAAAAEENTLISLCPEDDGDVPDMPFFAASNLEDRPVAPAAEDYPLGCEVEL      58
KAG5474614.1_L.ori               MQMFLD--VAAAAEENTLISFCPEDDGDVPDMPFFAASNLEDRPVAPAAEDYPLGCEVEL      58
XP_001566084.1_L.bra             MHMSLG--VADATAEGNLFSLCPEDDGNVPDMPFFAASNLEDRPIAPAAEDYPLGCEVEL      58
XP_010700392.1_L.pan             MHMSLG--VADATAEGNLFSLCPEDDGNVPDMPFFAASNLEDRPIAPAAEDYPLGCEVEL      58
XP_003876883.1_L.mex             MQMFMD--IAAAAEEDNLISFCPEDDGDVPDMPFFAASNLDDRPIAPAAEDYPLGCEVEL      58
XP_001684328.1_L.maj             MQMFMD--IAAAAEEDNLVSLCPEDDGDVPDMPFFAASNLDDRPIAPAAEDYPLGCEVEL      58
XP_001470086.1_L.inf             MQMFMG--IAAAAEEDNLISLCPEDDGDVPDMPFFAASNLDDRPIAPAAEDYPLGCEVEL      58
TPP40581.1_TPR_repeat_L.don      MQMFMG--IAAAAEEDNLISLCPEDDGDVPDMPFFAASNLDDRPIAPAAEDYPLGCEVEL      58
XP_003862152.1_L.don             MQMLMG--IAAAAEEDNLISLCPEDDGDVPDMPFFAASNLDDRPIAPAAEDYPLGCEVEL      58
                                 *:* :.   * *: *..*.*:******:************:***:***************

LSCM1_03062_L.mar                RDVEFAKIAQESSHWEPIMEAVFAVPRRRVRVDRHIGDYTFVLCNDIDGFERVGCTLYRA      120
KAG5474951.1_L.enr               RDIEFAKIAQESSHWEPIMEVVFAVPRRRVRVDRHTGDYTFVLCNDIDGFELVGCTLYRA      118
KAG5498199.1_L.Nam               RDVEFAKIAQESSHWEPIMEAVFAVPRRRVRVDRHTGDYTFVLCNDIDGFERVGCTLYRA      118
KAG5499776.1_L.Gha               RDIEFAKIAQESSHWEPIMEAVFAVPRRRVRVDRHTGDYTFVLCNDIDGFERVGCTLYRA      118
KAG5474614.1_L.ori               RDIEFAKIAQESSHWEPIMEAVFAVPRRRVRVDRHTGDYTFVLCNDIDGFERVGCTLYRA      118
XP_001566084.1_L.bra             RDIEFAKIAQESSHWEPIMEAVFAVPRRRVRVDRHTGDYTFVLCNDIDGFERVGCTLYRA      118
XP_010700392.1_L.pan             RDIEFAKIAQESSHWEPIMEAVFAVPRRRVRVDRHTGDYTFVLCNDIDGFERVGCTLYRA      118
XP_003876883.1_L.mex             RDVEFAKIAQESSHWEPIMEVVFAVPRRRVQVDRHTGDYTFVLCNDIDGFERVGCTLYRA      118
XP_001684328.1_L.maj             RDVEFAKIAQESSHWEPIMEAVFAVPRRRVRVDRHTGDYTFVLCNDIDGFERVGCTLYRA      118
XP_001470086.1_L.inf             RDVEFAKIAQESSHWEPIMEAVFAVPRRRVRVDRHTGDYTFVLCSDIDGFERVGCTLYRA      118
TPP40581.1_TPR_repeat_L.don      RDVEFAKIAQESSHWEPIMEAVFAVPRRRVRVDRHTGDYTFVLCSDIDGFERVGCTLYRA      118
XP_003862152.1_L.don             RDVEFAKIAQESSHWEPIMEAVFAVPRRRVRVDRHTGDYTFVLCSDIDGFERVGCTLYRA      118
                                 **:*****************.*********:**** ********.****** ********

LSCM1_03062_L.mar                CLSEPKVKATRYYSSIRSGISGLELDGNAEDIVRKPGQLGAATITAAEAIGTVVEARVVA      180
KAG5474951.1_L.enr               CLSEPKVKATRYYSSIRSGLSRLDLDGNAEDIARKPGPLGAATNTAAEAVSTVVDAREAA      178
KAG5498199.1_L.Nam               CLSEPKVKATRYYSSIRSGLSRLELDGNAEDIVRKPGPLGAATNTAAEAVSTVVEAREAA      178
KAG5499776.1_L.Gha               CLSEPKVKATRYYSSIRSGLSRLELDGNAEDIVRKPGPLGAAKNTAAEAVSTVVAAREAA      178
KAG5474614.1_L.ori               CLSEPKVKATRYYSSIRSGLSRLELDGNAEDIVRKPGPLGAAANTAAEAVSTVVEAREAA      178
XP_001566084.1_L.bra             CLSEPKVKATRYYSSIRSALSGLELEDNAEDILSRPVQLCVATVTAAETGCTVLEARGAA      178
XP_010700392.1_L.pan             CLSEPKVKATRYYSSIRSALSGLELEDNAEDILSRPVQLCVATATAAETGCTVLEARGAA      178
XP_003876883.1_L.mex             CLSEPKVKATRYYSSIRSGLSGLELEDNAEEILSKPVQLGAVTSTAAEANRTVMDARVAA      178
XP_001684328.1_L.maj             CLSEPKVKATRYYSSIRSGLSGLELEDNGEEILSKPAQLGAATSAAAEANKTVMDTRVAA      178
XP_001470086.1_L.inf             CLSEPKVKATRYYSSIRSGLSGLELEDNAEEILSKPVQLGAATSTAAEANRTVMDARVAA      178
TPP40581.1_TPR_repeat_L.don      CLSEPKVKATRYYSSIRSGLSGLELEDNAEEILSKPVQLGAATSTAAEANRTVMDARVAA      178
XP_003862152.1_L.don             CLSEPKVKATRYYSSIRSGLSGLELEDNAEEILSKPVQLGAATSTAAEANRTVMDARVAA      178
                                 ******************.:* *:*:.*.*:*  :*  * ..  :***:  **: :* .*

LSCM1_03062_L.mar                TASAGNSGVLTSLKSLRSAHSMGSLSDEARRRSFMAYMRKGTAEIGKRNCEAALRFYNRA      240
KAG5474951.1_L.enr               TVSAGKCGVPASVKSVRSAHNMGSSSDEARRRSFMAYMRKGTAEIGKRNCEAALRYYNRA      238
KAG5498199.1_L.Nam               TVSAGKCGVPTSVKSVRSTHNMGNSSDEARRRSFMAYMRKGTAEIGKRNCEAALRYYNRA      238
KAG5499776.1_L.Gha               TVSAGKCGVPASVKSVRSAYNMGNSSDEARRRSFMAYMRKGTAEIGKRNCEAALRYYNRA      238
KAG5474614.1_L.ori               TVSAGKCGVPTSVKSVRNAHTMGNSSDEARRRSFMAYMRKGTAEIGKRNCEAALRYYNRA      238
XP_001566084.1_L.bra             TPSAGNNSILTSVKSVRSAVYMTSSADDARRRSFMTYMRRGTAEIGKRNCEAALRYYNRA      238
XP_010700392.1_L.pan             TPSAGNNNILTSVKSVRSAVYMTSSADDARRRSFMTYMRRGTAEIGKRNCEAALRYYNRA      238
XP_003876883.1_L.mex             TASTGNNGV-ASLKSVRSAANVAGSADDARRRSFMAYMKKGTAEIGKRNCEAALRYYNRA      237
XP_001684328.1_L.maj             TASAGNNRV-ASLKSVRSAASVASSADDARRRSFMAYMKKGTAEIGKRNCEAALRYYNRA      237
XP_001470086.1_L.inf             TASTGNNRV-ASLKSVRGAANVASSADDARRRSFMAYMKKGTAEIGKRNCEAALRYYNRA      237
TPP40581.1_TPR_repeat_L.don      TASTGNNRV-ASLKSVRGAANVASSADDARRRSFMAYMKKGTAEIGKRNCEAALRYYNRA      237
XP_003862152.1_L.don             TASTGNNRV-ASLKSVRGAANVASSADDARRRSFMAYMKKGTAEIGKRNCEAALRYYNRA      237
                                 * *:*:  : :*:**:*.:  : . :*:*******:**::***************:****

LSCM1_03062_L.mar                LEEMPEEEARVRSSRSVAYLLTGEKERALEDANRVVHLLPRESVGYIRTGNVLRSMKKFV      300
KAG5474951.1_L.enr               LEEMPEEEARVRSSRSVAYLLAGEKERALEDANRVVHLVPWESVGYIRTGNVLRSMKKFV      298
KAG5498199.1_L.Nam               LEEMPEEEARVRSSRSVAYLLAGEKERALEDANRVVHLVPWESVGYIRTGNVLRSMKKFV      298
KAG5499776.1_L.Gha               LEEMPEEEARVRSSRSVAYLLAGEKERALEDANRVVHLVPWESVGYIRTGNVLRSMKKFV      298
KAG5474614.1_L.ori               LEEMPEEEARVRSSRSVAYLLAGEKERALEDANRVVHLVPWESVGYIRTGNVLRSMKKFV      298
XP_001566084.1_L.bra             LEEMPEEEARVRSSRSVAYLLAGEKDRALEDANRVVHLVPRESVGYIRTGNVLRSMKKFV      298
XP_010700392.1_L.pan             LEEMPEEEARVRSSRSVAYLLAGEKDRALEDANRVVHLVPRESVGYIRTGNVLRSMKKFV      298
XP_003876883.1_L.mex             LEEMPEEEARVRSSRSVAYLLAGEKERALEDANRVVHLVPRESVGYIRTGNVLRSMKKFV      297
XP_001684328.1_L.maj             LEEMPEEEARVRSSRSVAYLLAGEKDRALEDANRVVHLVPRESVGYIRTGNVLRSMKKFV      297
XP_001470086.1_L.inf             LEEMPEEEARVRSSRSVAYLLAGEKDRALEDANRVVHLVPRESVGYIRTGNVLRSMKKFV      297
TPP40581.1_TPR_repeat_L.don      LEEMPEEEARVRSSRSVAYLLAGEKDRALEDANRVVHLVPRESVGYIRTGNVLRSMKKFV      297
XP_003862152.1_L.don             LEEMPEEEARARSSRSVAYLLAGEKDRALEDANRVVHLVPRESVGYIRTGNVLRSMKKFV      297
```

```
                                **********.**********:***:************:* *******************

LSCM1_03062_L.mar               DAQKMYATALKFDSSSAVVRNLLEGNSVAMLYEGRTKRYKKMRIVYDSSMKRAIAVSAQN      360
KAG5474951.1_L.enr              DAQKMYATALKYDSSSTVVRNLLEGNSVAMLYEGRTKRYKKMRIVFDNPTKRAIAVSAQN      358
KAG5498199.1_L.Nam              DAQKMYATALKYDSSSTVVRNLLEGNSVAMLYEGRTKRYKKMRIVFDNSTKRAIAVSAQK      358
KAG5499776.1_L.Gha              DAQKMYATALKYDSSSTVVRNLLEGNSVAMLYEGRTKRYKKMRIVFDNSTKRAIAVSAQN      358
KAG5474614.1_L.ori              DAQKMYATALKYDSSSTVVRNLLEGNSVAMLYEGRTKRYKKMRIVFDNSTKRAIAVSAQN      358
XP_001566084.1_L.bra            DAQKMYATALKYDSSSAVVRILLEGNSVAMLYEGRTKRYKKMRIFYDDAAQRAIAVAAQN      358
XP_010700392.1_L.pan            DAQKMYATALKYDSSSTVVRILLEGNSVAMLYEGRTKRYKKMRIFYDDAAQRAIAVAAQN      358
XP_003876883.1_L.mex            DAQKMYATALKCDSASAVVRNLLEGNSVAMLYEGRTKRYKKMRIAYDDSMKRAIAVVAQN      357
XP_001684328.1_L.maj            DAQKMYATALKYDSSSAVVRNLLEGNSVAMLYEGRTKRYKKMRIAYDDSMKRAIAVVAQS      357
XP_001470086.1_L.inf            DAQKMYATALKYDSSSAVVRNLLEGNSVAMLYEGRTKRYKKMRIAYDDSMRRAIAVVAQN      357
TPP40581.1_TPR_repeat_L.don     DAQKMYATALKYDSSSAVVRNLLEGNSVAMLYEGRTKRYKKMRIAYDDSMRRAIAVVAQN      357
XP_003862152.1_L.don            DAQKMYATALKYDSSSAVVRNLLEGNSVAMLYEGRTKRYKKMRIAYDDSMRRAIAVVAQN      357
                                *********** **:*:*** ********************** :*.  :***** **.

LSCM1_03062_L.mar               IDANEIVMKEASSCVFPLSCGIYRVVSGRQRCPMCCHCGRPFVDEAQVTEQIPGLTKQLL      420
KAG5474951.1_L.enr              IDVNEIVMKEASSCVFPLSSGIYRVVSGRQRCPMCCHCGRPFVDEARVTEQIPGLTKQLL      418
KAG5498199.1_L.Nam              IDADEIVMKEASSCVFPLSSGIYRVVSGRQRCSMCCHCGRPFVDEAQVTEQIPGLTKQLL      418
KAG5499776.1_L.Gha              IDANEIVMKEASSCVFPLSSGIYRIVSGRQRCPMCCHCGRPFVDEDQVTEQIPGLTKQLL      418
KAG5474614.1_L.ori              VDVNEIVMKEATSCVFPLSSGIYRVVSGRQRCPMCCHCGRPFVDEAQVTEQIPGLTKQLL      418
XP_001566084.1_L.bra            MDANEIVMKEASSCVFPLLSGIYRVASGRQCCPMCCHCGRPFVDEAQMAEQIPGLTKQLL      418
XP_010700392.1_L.pan            MDASEIVMKEASSCVFPLLSGIYRVASGRQCCPMCCHCGRPFVDEAQMAEQIPGLTKQLL      418
XP_003876883.1_L.mex            IDANEIVMKEATSCVFPLLSSIYRVVNGRQRCPMCCHCGRPFVDEAQVTEQIPGLTKHLL      417
XP_001684328.1_L.maj            IDANEIVMKEGTSCVFPLSSGIYRIVNGRQRCPMCCHCGRPFVDEAQVTEQIPGLTKQLL      417
XP_001470086.1_L.inf            IDANEIVMKEATSCVFPLSSGIYRVVNGRQRCPMCCHCGRPFVDEAQVTEQIPGLTKQLL      417
TPP40581.1_TPR_repeat_L.don     IDANEIVMKEATSCVFPLSSGIYRVVNGRQRCPMCCHCGRPFVDEAQVTEQIPGLTKQLL      417
XP_003862152.1_L.don            IDANEIVMKEATSCVFPLSSGIYRVVNGRQRCPMCCHCGRPFVDEAQVTEQIPGLTKQLL      417
                                :*..******.:****** ..***:..*** * ************ :::********:**

LSCM1_03062_L.mar               SSLQVVVHPVPCDSDCNAFYCSESCKSKAWVEHHWVECTQRGRWREGVPVVHRLLDQYVT      480
KAG5474951.1_L.enr              SSLQVVVNPVPCDSNCNAFYCSESCKSKAWVEHHWVECTQRGRWREGVPVVHRLLDQYVA      478
KAG5498199.1_L.Nam              SSLQVVVNPVPCDSNCNAFYCSENCKSKAWVEHHWVECTQRGRWREGVPVVHRLLDQYVA      478
KAG5499776.1_L.Gha              SSLQVVVNPVPCDSNCNAFYCSESCKSKAWVEHHWVECTQRGRWREGVPVVHRLLDQYVA      478
KAG5474614.1_L.ori              SSLQVLVNPVPCDSNCNAFYCSESCKSKAWVEHHWVECTQRGRWREGVPVVHCLLDQYVA      478
XP_001566084.1_L.bra            NSLHVVVRPVPCDSSCNALYCSESCKSKAWVEHHWVECTQRGRWRDGVPVVHCLLDQYVM      478
XP_010700392.1_L.pan            NSLHVVVRPVPCDSSCNALYCSESCKSKAWVEHHWVECTQRGRWRDGVPVVHCLLDQYVM      478
XP_003876883.1_L.mex            NSLHVLVNPVPCDSNCSAFYCSENCKSKAWVEHHWVECTQRGRWRDGVPVVHRLLDQFVT      477
XP_001684328.1_L.maj            NSLHVLVNPVPCDSNCSALYCSEGCKSKAWVEHHWVECTQRGRWRDGVPVVHRLLDQFVT      477
XP_001470086.1_L.inf            NSLHVLVNPVPCDSNCSALYCSESCKSKAWVEHHWVECTQRGRWRDGVPVVHRLLDQFVT      477
TPP40581.1_TPR_repeat_L.don     NSLHVLVNPVPCDSNCSALYCSESCKSKAWVEHHWVECTQRGRWRDGVPVVHRLLDQFVT      477
XP_003862152.1_L.don            NSLHVLVNPVPCDSNCSALYCSESCKSKAWVEHHWVECTQRGRWRDGVPVVHRLLDQFVT      477
                                .**:*:*.******.*.*:****.*********************:****** ****:*

LSCM1_03062_L.mar               ACSACALAADELSDASPAPKCVAEDTRPLITAACVRIAVRMMSRMVSSVLPAQEAVQQYR      540
KAG5474951.1_L.enr              ACSTCASAVEESNDASPAPKCVAEDTRPPITAACVRIAVRMMSRMVSSVLPAQEAVQQYR      538
KAG5498199.1_L.Nam              ACSTCASAVEEPNDASPAPKCVAEDTRPLITAACVRIAVRMMSRMVSSVLPAQEAVQQYR      538
KAG5499776.1_L.Gha              ACSTCTSAVEEPNDASPAPKCVAEDTRPLITAACVRIAVRMMSRMVSSVLPAQEAVQQYR      538
KAG5474614.1_L.ori              ACSTCASAVEEPNDASPAPKCVAEDTRPLITAACVRIAVRMMSRMVSSVLPAQEAVQQYR      538
XP_001566084.1_L.bra            TCAANAADEEVPTDISPMPQCVAEDTRPIITAACVRIAVRMMSRMVSSVLPAQEAVQQYR      538
XP_010700392.1_L.pan            TCAANAADEEVPTDISPTPQCVAEDTRPIITAACVRIAVRMMSRMVSSVLPAQEAVQQYR      538
XP_003876883.1_L.mex            ARSANSPVEETPCAVSPAPQCVAEDTRPIITAACVRIAVRMMSRMVSSVLPAQEAVQQYR      537
XP_001684328.1_L.maj            TRSARSFVEDAPCDVSPAPQCVAEDTRPIIIAACVRIAVRMMSRMVSSVLPAQEAVQQYR      537
XP_001470086.1_L.inf            TCSANSAAEEAPCDVSPAPQCVAEDTRPIITAACVRIAVRMMSRMVSSVLPAQEAVQQYR      537
TPP40581.1_TPR_repeat_L.don     TCSANSAAEEAPCDVSPAPQCVAEDARPIITAACVRIAVRMMSRMVSSVLPAQEAVQQYR      537
XP_003862152.1_L.don            TCSANSAAEEAPCDVSPAPQCVAEDTRPIITAACVRIAVRMMSRMVSSVLPAQEAVQQYR      537
                                : :: :   :     ** *:*****:** * ****************************

LSCM1_03062_L.mar               WLPVADVVRSQRREVKELLMTCYATLSCGFSSEDSDQLTFEVFQEYYEKAKSNSIMICCS      600
KAG5474951.1_L.enr              WLPVADVVRGQRREVKELLTRCYSTLSCGFSSEDSDQLTFEVFQEYYEKAKSNSIMICCS      598
KAG5498199.1_L.Nam              WLPVADVVRGQRREVKELLTRCYSTLSCGFSSEDSDQLTFEVFQEYYEKAKSNSIMICCS      598
KAG5499776.1_L.Gha              WLPVADVVRGQRREVKELLTRCYSTLSCGFSSEDSDQLTFEVFQEYYEKAKSNSIMICCS      598
KAG5474614.1_L.ori              WLPVADVVRGQRREVKELLKRCYSTLSCGFSSEDSDQLTFEVFQEYYEKAKSNSIMICCS      598
XP_001566084.1_L.bra            WLPVADVVRSQRREVKELLITCYSTLSQGFSSEDSDQLTFEVFQDYYEKAKSNSIIICCS      598
XP_010700392.1_L.pan            WLPVADVVRSQRREVKELLITCYSTLSQGFSSEDSDQLTFEVFQDYYEKAKSNSIIICCS      598
XP_003876883.1_L.mex            WLPIANVVRGQRRQVKELLMTCYSTLSSGFSSEDSDQLTFEVFQEYYEKAKSNSIMICCS      597
XP_001684328.1_L.maj            WLPIADVVRGQRREVKELLMTCYSTLSRGFSSEDSDQLTFKVFQEYYEKAKSNSIMICCS      597
XP_001470086.1_L.inf            WLPIADVVRGQRREVKELLRTCYSTLSRGFSSEDSDQLTFEVFQEYYEKAKSNSIMICCS      597
TPP40581.1_TPR_repeat_L.don     WLPIADVVRGQRREVKELLRTCYSTLSRGFSSEDSDQLTFEVFQEYYEKAKSNSIMICCS      597
XP_003862152.1_L.don            WLPIADVVRGQRREVKELLRTCYSTLSRGFSSEDSDQLTFEVFQEYYEKAKSNSIMICCS      597
                                ***:*:***.***:*****  **:*** ***********:***:*********:****

LSCM1_03062_L.mar               MWPKVVERVRGHLDLVTAEDSPAGMHDSEAPFHLCLQQIMNAPRQGVPGTYHICISMFEL      660
KAG5474951.1_L.enr              MWPKVVERVSSHLDLVTAEDSPAGMHDSEAPLHLCLHQIMNAPRQGVPGTYHICISMFEL      658
KAG5498199.1_L.Nam              MWPKVVERVSSHLDLVTAEDSPAGMHDSEAPLHLCLHQIMNAPRQGVPGTYHICISMFEL      658
KAG5499776.1_L.Gha              MWPKVVERVSSHLDLVTAEDSPAGMHDSEAPLHLCLHQIMNAPRQGVPGTYHICISMFEL      658
KAG5474614.1_L.ori              MWPKVVERVSSHLDLVTAEDSPAGMHDSEAPLHLCLHQIMNAPRQGVPGTYHICISMFEL      658
XP_001566084.1_L.bra            MWPKVVERVRSHLDLVTAEDSPAGLQDSDTPFHLCLQQVLNAPRQGVPGTYHICISMFEL      658
XP_010700392.1_L.pan            MWPKVVERVRSHLDLVTAEDSPAGLQDSDTPFHLCLQQVLNAPRQGVPGTYHICISMFEL      658
XP_003876883.1_L.mex            VWPKVVERVSSHLDFVTAEDSPAGLHGNEASFHLCMEQIMNAPRQGVPGTYHICISMFEL      657
XP_001684328.1_L.maj            VWPKVVERVSSHLDLVTAEDSPAGLHGTEASFDLCLQQIMNAPRQGVPGTYHICISMFEL      657
XP_001470086.1_L.inf            VWPKVVERVSSHLDLVTAEDSPAGLHGTEASFHLCLQQIMNAPRQGVPGTYHICISMFEL      657
TPP40581.1_TPR_repeat_L.don     VWPKVVERVSSHLDLVTAEDSPAGLHGTEASFHLCLQQIMNAPRQGVPGTYHICISMFEL      657
XP_003862152.1_L.don            VWPKVVERVSSHLDLVTAEDSPAGLHGTEASFHLCLQQIMNAPRQGIPGTYHICISMFEL      657
                                :******** .***:********::..:: :.**:.*::******:************

LSCM1_03062_L.mar               ASLMTSNNCTRSETLRPNVMVRTFAESAASLRLRTLRPITKEEMLTMTWPAYDAA-         715
KAG5474951.1_L.enr              ASLMMSNNCTRSEAMRPNVMVRTFAESAANLRLRTLRPIAKEEMLTMTWPAYGPA-         713
```

```
KAG5498199.1_L.Nam              ASLMMSNNCTRSETMRPNVMVRTFAESAANLRLRTLRPIAKEEMLTMTWPAYDPA-         713
KAG5499776.1_L.Gha              ASLMMSNNCTRSETMRPNVMVRTFAESAANLRLRTLRPIAKEEMLTMTWPAYDPA-         713
KAG5474614.1_L.ori              ASLMMSNNCTRSETMRPNVMVRTFAESAANLRLRTLRPIAKEEMLTMTWPAYDPAS         714
XP_001566084.1_L.bra            ASLMTSNNCTRSETMRPNVMVRTFAESAASLRLRTLRPIAKEEVLTMDWPVYGAA-         713
XP_010700392.1_L.pan            ASLMTSNNCTRSETMRPNVMVRTFAESAASLRLRTLRPIAKEEVLTMDWPVYGAA-         713
XP_003876883.1_L.mex            ASLMTSNNCTRSEVMRPNVMVRTFAESAANLRLRTLRPVAKEEVLTMAWPAYGAA-         712
XP_001684328.1_L.maj            ASLMTSNNCTRSEVMRPNVMVRTFAESAANLRLRTLRPVAKEEVLAMAWPAYGAA-         712
XP_001470086.1_L.inf            ASMMTSNNCTRSEVMRPNVMVRTFAESAANLRLRTLRPVAKEEVLTMAWPAYGAA-         712
TPP40581.1_TPR_repeat_L.don     ASLMTSNNCTRSEVMRPNVMVRTFAESAANLRLRTLRPVAKEEVLTMAWPAYGAA-         712
XP_003862152.1_L.don            ASLMTSNNCTRSEVMRPNVMVRTFAESAANLRLRTLRPVAKEEVLTMAWPAYGAA-         712
                                **:* ********.:**************.********::***:*:* **.*. *
```

Fig. S2 Blastp result of hypothetical protein LSCM1_03062 [*Leishmania martiniquensis*].
Description: hypothetical protein LSCM1_03062 [Leishmania martiniquensis]...
Molecule type: amino acid
Query Length: 715

Descriptions | Graphic Summary | Alignments | Taxonomy

Sequences producing significant alignments — Download | Select columns | Show 100

select all *100 sequences selected* — GenPept | Graphics | Distance tree of results | Multiple alignment | MSA Viewer

Description	Scientific Name	Max Score	Total Score	Query Cover	E value	Per. Ident	Acc. Len	Accession
hypothetical protein LSCM1_03062 [Leishmania martiniquensis]	Leishmania mart...	1480	1480	100%	0.0	100.00%	715	KAG5474282.1
hypothetical protein JIQ42_03003 [Leishmania sp. Namibia]	Leishmania sp. ...	1327	1327	100%	0.0	91.47%	713	KAG5498199.1
hypothetical protein GH5_03911 [Leishmania sp. Ghana 2012 LV757]	Leishmania sp. ...	1324	1324	100%	0.0	91.05%	713	KAG5499776.1
hypothetical protein LSCM4_03787 [Leishmania orientalis]	Leishmania orie...	1310	1310	100%	0.0	90.91%	713	KAG5474614.1
hypothetical protein CUR178_04401 [Leishmania enriettii]	Leishmania enri...	1307	1307	100%	0.0	90.49%	713	KAG5474951.1
conserved hypothetical protein [Leishmania mexicana MHOM/GT/2001/U1103]	Leishmania mex...	1278	1278	100%	0.0	85.87%	712	XP_003876883.1
conserved hypothetical protein [Leishmania infantum JPCM5]	Leishmania infa...	1269	1269	100%	0.0	86.99%	712	XP_001470086.1
hypothetical protein, conserved [Leishmania donovani]	Leishmania don...	1268	1268	100%	0.0	86.85%	712	XP_003862152.1
TPR repeat family protein [Leishmania donovani]	Leishmania don...	1268	1268	100%	0.0	86.99%	712	TPP40581.1
conserved hypothetical protein [Leishmania braziliensis MHOM/BR/75/M2904]	Leishmania braz...	1228	1228	100%	0.0	85.59%	713	XP_001566084.1
conserved hypothetical protein [Leishmania major strain Friedlin]	Leishmania maj...	1227	1227	100%	0.0	86.29%	712	XP_001684328.1
hypothetical protein LPMP_280510 [Leishmania panamensis]	Leishmania pan...	1224	1224	100%	0.0	85.31%	713	XP_010700392.1
hypothetical protein, conserved [Leishmania tarentolae]	Leishmania tare...	1204	1204	100%	0.0	83.08%	711	GET89968.1
hypothetical protein JKF63_03467 [Porcisia hertigi]	Porcisia hertigi	1090	1090	98%	0.0	77.49%	712	KAG5500375.1

Fig. S3 Alignment of the protein sequences corresponding to the hypothetical protein LSCM1_02556 [*Leishmania martiniquensis*]. From top to bottom parasites listed are *L.* (*V.*) *braziliensis*, *L.* (*V.*) *panamensis*, *L.* (*V.*) *guyanensis*, *L.* (*M.*) *martiniquensis*, *L.* (*M.*) *enriettii*, *L.* sp. *Namibia* (*L.* (*M.*) *procaviensis* n. sp.), *L.* sp. *Ghana* (*L.* (*M.*) *chancei* n. sp.), *L.* (*M.*) *orientalis*, (*L.*) *donovani*, *L.* (*L.*) *infantum*, *L.* (*L.*) *mexicana*. Proteins sequences are from Blastp (https://blast.ncbi.nlm.nih.gov/Blast.cgi). Alignment was performed using Clustal Omega (https://www.ebi.ac.uk/jdispatcher/msa/clustalo). Note: The LSCM1_02556 sequence is similar to the sterol C-24 reductase sequences of other species.

```
CLUSTAL O(1.2.4) multiple sequence alignment


XP_001567842.1_L.bra      MPENNRSSNHVAPRSRSRSRSRTRKASRTRSNTPRRTKVLTPEEAYYATVERKFTPEKDE 60
XP_010702166.1_L.pan      MPENNRSSNHVAPRSRSRSRSRTRKASRTRSNTPRRTKVLTPEEAYYATVERKFTPEKDE 60
CCM18528.1_L.guy          MPENNRSSNHVAPRSRSRSRSRTRKASRTRSNTPRRTKVLTPEEAYYATVERKFTPEKDE 60
LSCM1_02556_L.mar         MSSNKRST----SRAASRSRSRSRKGSRARSSTPSRPKALTPEEAYHATVERKFTPEKDA 56
KAG5469688.1_L.enr        MPSNKRST----SRAASRSRSRSRKASRTRSSTPSRAKALTPQEAYYATVERKFTPEKDP 56
KAG5493492.1_L.Nam        MPSNKRST----SRAASRSRSRSRKASRTRSSTPSRAKALTPEEAYYATVERKFTPEKDP 56
KAG5494252.1_L.Gha        MPSNKRST----SRAASRSRSRSRKASRTRSSTPSRAKALTPQEAYYATVERKFTPEKDP 56
KAG5468988.1_L.ori        MPSNKRST----SRAASRSRSRSRKASRTRSSTPSRARALTPQEAYYATVERKFTPEKDP 56
XP_003863897.1_L.don      MPTSKKSA----SRAASRSRSRSRKASRARSRTPSRARVLTPEEAYYAPVERKFTPEKDV 56
XP_001468153.1_L.inf      MPTSKKSA----SRAASRSRSRSRKASRARSRTPSRARVLTPEEAYYAPVERKFTPEKDV 56
XP_003878317.1_L.mex      MPTSKKSA----SRAASRSRSRSRKTSRPRSRTPSRSRALTPEEAYYATVERKFTPEKDK 56
                          *  .::*:     *: ******:** ** ** ** * :.***:***:* **********

XP_001567842.1_L.bra      WNEEWEFQGPLGVLLIMAVSHVLIFYFYVCVEKFQGTIIYPGHPKLEGKKMQTVFFSFLA 120
XP_010702166.1_L.pan      WNEEWEFQGPLGVLFIMAVSHVLIFYFYVCVERFQGAIIYPGHPKLEGEKMQTVFFSFLA 120
CCM18528.1_L.guy          WNEEWEFQGPLGVLFIMAVSHVLIFYFYVCVERFQGAIIYPGHPKLEGEKMQTVFFSFLA 120
LSCM1_02556_L.mar         WRGNWEFNGPVGVLAIMALSHVLIFYFYVCIEEFKGTLIYPGHHMLRGEKMQTVFLSFLA 116
KAG5469688.1_L.enr        WHGNWEFNGPIGVLGIMVVSHVLIFYFYVCIEKFQGALIYPRHPMLKGERMQTVFFSFLA 116
KAG5493492.1_L.Nam        WRGNWEFNGPVGVLGIMVVSHVLIFYFYVCIEKFEGTLIYPRHPMLKGERMQTVFFSFLA 116
KAG5494252.1_L.Gha        WRGNWEFNGPVGVLGIMVVSHVLIFYFYVCIEKFQGTLIYPRHPMLKGERMQTVFFSFLA 116
KAG5468988.1_L.ori        WRGNWEFNGPVGVLGIMVVSHVLIFYFYVCIEKFQGTLIYPRHPMLKGERMQTVFFSFLA 116
XP_003863897.1_L.don      WRGNWEFNGPLGVLGIMIVSHVIIYYFYVCIELFQGTIIYPGHPMLKGEKMQKVFFSFLA 116
XP_001468153.1_L.inf      WRGNWEFNGPLGVLGIMIVSHVIIYYFYVCIELFQGTIIYPGHPMLKGEKMQKVFFSFLA 116
XP_003878317.1_L.mex      WHGNWEFNGPLGVSAIMIASHVLIYYFYVCIELFQGTIIYPGHPMLKGEKMQKVFFSFLA 116
                          *. :***:**:**  **  ***:*:*****:* *:*::*** *  *.*::**.**:****

XP_001567842.1_L.bra      EHACPTVRTFVIFLGFLLLEYFLALVLPAIYVKGLPLPSENGYRLTYKCNAISAWYCILV 180
XP_010702166.1_L.pan      EHACPTVRTFVIFLGFLLLEYFLALVLPAIYVKGLPLPSENGYRLTYKCNAVSAWYCILV 180
CCM18528.1_L.guy          EHACPTVRTFVIFLGFLLLEYFLALVLPAIYVKGLPLPSENGYRLTYKCNAVSAWYCILV 180
LSCM1_02556_L.mar         EHACPTFGSAAVFLGFLLLEYVLALMLPAIYVKGLPLPSENGYRLTYKCNAVSAWYCVLI 176
KAG5469688.1_L.enr        EHACPTAEAFGIFLGFLLLEYFLALVLPALYVKGLPLPSENGYRLTYKCNAVSAWYCVVI 176
KAG5493492.1_L.Nam        EHACPTAEAFGIFLGFLLLEHFLALVLPALYVKGLPLPSENGYRLTYKCNAVSAWYCVLI 176
KAG5494252.1_L.Gha        EHACPTAEALGIFLGFLLLEYFLALVLPALYVKGLPLPSENGYRLTYKCNAVSAWYCVLI 176
KAG5468988.1_L.ori        EHACPTAEALGIFLGFLLLEYFLALVLPALYVKGLPLPSENGYRLTYKCNAVSAWYCVLI 176
XP_003863897.1_L.don      EHACPTAQTFCIFLGFLLLEYLLALVLPALYVKGLPLPSENGYRLTYKCNAVTAWYCLLI 176
XP_001468153.1_L.inf      EHACPTAQTFCIFLGFLLLEYLLALVLPALYVKGLPLPSENGYRLTYKCNAVTAWYCLLI 176
XP_003878317.1_L.mex      EHACPTVQSFCIFLGFLLLEYFLALVLPALYVKGLPLPSENGYRLTYKCNAVSAWYCMFI 176
                          ******  :  :********:.***:***:*********************::****:.:

XP_001567842.1_L.bra      IVGFLHYYGVYPLNELRRNYGHYLTVATITADVISVWVYVAGYKRRIRMTGNFIYDFFMG 240
XP_010702166.1_L.pan      IVGFLHYYGIYPLNELRRNYGHYLTVATITADVISVWVYIAGYKRRIRMTGSFIYDFFMG 240
CCM18528.1_L.guy          IVGFLHYYGVYPLNELRRNYGHYLTVATITADVISVWVYIAGYKRRIRMTGSFIYDFFMG 240
LSCM1_02556_L.mar         IVGLLHYYGIYPLNELRRNYGHFLTTATITADFISVWVYVTGYKRRIRMTGNFIYDFFMG 236
KAG5469688.1_L.enr        IVGLLHYYGVYPLNELRRNYGHFLSVATIVADIIAVWVYVAGYKRRIRMTGNFIYDFFMG 236
KAG5493492.1_L.Nam        LVGLLHYYGVYPLNELRRNYGHFLSVATIVADIIAVWAYIAGYKRRIRMTGNFIYDFFMG 236
KAG5494252.1_L.Gha        IVGLLHYYGVYPLNELRRNYGHFLSVATIVADIIAVSVYIAGYKRRIRMTGNFIYDFFMG 236
KAG5468988.1_L.ori        IVGLLHYYGVYPLNELRRNYGHFLSVATIVADIIAVSVYIAGYKRRIRMTGNFIYDFFMG 236
XP_003863897.1_L.don      IVGLLHYYGVYPINELRRNYGHFLTVATIVADIIAVWVYIAGYKRRIRMTGNFIYDFFMG 236
XP_001468153.1_L.inf      IVGLLHYYGVYPINELRRNYGHFLTVATIVADIIAVWVYIAGYKRRIRMTGNFIYDFFMG 236
XP_003878317.1_L.mex      IVGLLHYYGVYPVNELRRNYGHFLTVATIVADIIAVWVYIAGYKRRIRMTGNFIYDFFMG 236
                          :**:*****:**:*********:*:.***.**.*:* .*::**********.********

XP_001567842.1_L.bra      SALNLRLPGNIDVKMFAECRNSWVLLMLLTLSCAAEQYNELGYLTGNMIFMIMAHLLYVN 300
XP_010702166.1_L.pan      SALNLRLPGNIDVKMFAECRNSWVLLMLLTLSCAAEQYNELGYLTGNMIFMIMAHLLYVN 300
CCM18528.1_L.guy          SALNLRLPGNIDVKMFAECRNSWVLLMLLTLSCAAEQYNELGYLTGNMIFMIMAHLLYVN 300
LSCM1_02556_L.mar         SALNYRLPGNIDVKLFAECRNSWVLLMMLTLSCAAEQYRELGYLTGNMIFMILAHLLYVN 296
KAG5469688.1_L.enr        SSLNYRLPGNIDVKLFAECRNSWVLLMMLTLSCAAEQYEELGYLTGNMLFMILAHLLYVN 296
KAG5493492.1_L.Nam        SGLNYRLPGNIDLKLFAECRNSWVLLMMLTLSCAAEQYDELGYLTGNMLFMILAHLLYVN 296
KAG5494252.1_L.Gha        SALNYRLPGNIDVKLFAECRNSWVLLMMLTLSCAAEQYDELGYLTGNMLFMILAHLLYVN 296
KAG5468988.1_L.ori        SALNYRLPGNIDVKLFAECRNSWVLLMMLTLSCAAEQYDELGYLTGNMLFMILAHLLYVN 296
XP_003863897.1_L.don      SGLNFRLPGNIDVKLFAECRNSWVLLMMLTLSCAAEQYNELGYLTGNMVFMISAHLLYVN 296
XP_001468153.1_L.inf      SGLNFRLPGNIDVKLFAECRNSWVLLMMLTLSCAAEQYNELGYLTGNMVFMISAHLLYVN 296
XP_003878317.1_L.mex      SGLNFRLPGNIDVKLFAECRNSWVLLMMLTLSCAAEQYKELGYLTGNMTFMILAHLIYVN 296
                          *.** *******:*:************:********** ********* *** ***:***

XP_001567842.1_L.bra      AVAKGEECVITTWDIYYEKFGWMLAYWNTCGVPFLYCMQSVYIQTVLKEKEHPRWVLALM 360
XP_010702166.1_L.pan      AVAKGEECVITTWDIYYEKFGWMLAYWNTCGVPFLYCMQSVYIQTVLKEKEHPRWVLALM 360
CCM18528.1_L.guy          AVAKGEECVITTWDIYYEKFGWMLAYWNTCGVPFLYCMQSVYIQTVLKEKEHPRWVLALM 360
LSCM1_02556_L.mar         AVEKGEECIISTWDIAYEKFGWMLAYWNTCGVPFLYCMQAMYIQTVLKEKEHPRWILGLM 356
KAG5469688.1_L.enr        AVEKGEECIISTWDIYYEKFGWMLAYWNTCGVPFLYCMQSMYIQTVLKEKEHPRLVLVLM 356
```

```
KAG5493492.1_L.Nam        AVEKGEECIISTWDIYYEKFGWMLAYWNTCGVPFLYCMQSMYIQTVLKEKEHPRWVLVLM 356
KAG5494252.1_L.Gha        AVEKGEECIISTWDIYYEKFGWMLAYWNTCGVPFLYCMQSMYIQTVLKEKEHPRWVLVLM 356
KAG5468988.1_L.ori        AVEKGEECIISTWDIYYEKFGWMLAYWNTCGVPFLYCMQSMYIQTVLKEKEHPRWVLVLM 356
XP_003863897.1_L.don      AVEKGEECIITTWDIYYEKFGWMLAYWNTCGVPFLYCMQSMYIQTVLKKKEHPRWVLVLM 356
XP_001468153.1_L.inf      AVEKGEECIITTWDIYYEKFGWMLAYWNTCGVPFLYCMQSMYIQTVLKKKEHPRWVLVLM 356
XP_003878317.1_L.mex      AVEKGEECVISTWDIYYEKFGWMLAYWNTCGVPFLYCMQSMYIQTVLKKKEYPRWVLVLM 356
                          ** *****:*:**** ***********************::*******:**:** :* **

XP_001567842.1_L.bra      VCVLLLAYYLWDTTNSQKSHFRMRRSGVPMKIIRSTAFPQMPWCYIENPRTLKSATGELF 420
XP_010702166.1_L.pan      VCVLLLAYYLWDTTNSQKSHFRMRRSGVPMKIIRSTAFPQMPWCYIENPRTLKSATGELF 420
CCM18528.1_L.guy          VCVLLLAYYLWDTTNSQKSHFRMRRSGVPMKIIRSTAFPQMPWCYIENPRTLKSATGELF 420
LSCM1_02556_L.mar         VCILLAAYYLWDTINSQKNHFRMHRSGVPMEIIRSNAFPQMPWCYIENPRTLKSATGELF 416
KAG5469688.1_L.enr        VCILLLAYYLWDTINSQKNHFRMHRSGVPMEIIRNSAFPQMPWCYIENPRTLKSATGELF 416
KAG5493492.1_L.Nam        VCILLLAYYLWDTINSQKNHFRMHRSGVPMEIIRNSAFPQMPWCYIENPRTLKSATGELF 416
KAG5494252.1_L.Gha        VCILLLAYYLWDTINSQKNHFRMHRSGVPMEIIRNSAFPQMPWCYIENPRTLKSATGELF 416
KAG5468988.1_L.ori        VCILLLAYYLWDTINSQKNHFRMHRSGVPMEIIRNSAFPQMPWCYIENPRTLKSATGELF 416
XP_003863897.1_L.don      ACILLLAYYLWDAINSQKNHFRMRRSGVPMEIIRNNAFPQVPWCYIENPRTLKSATGELF 416
XP_001468153.1_L.inf      ACILLLAYYLWDAINSQKNHFRMRRSGVPMEIIRNNAFPQVPWCYIENPRTLKSATGELF 416
XP_003878317.1_L.mex      VCILLLAYYLWDAINSQKNHFRMRRSGVPMEIIRNTAFPQVPWCYVENPRMLKSATGELF 416
                          .*:** ******: ****.****:******:***..****:****:**** *********

XP_001567842.1_L.bra      VDGFYRYGRKLHYTADLVMALLWGCACGFKSFIPFFYFSFFLTHLIHRERRDEHRCKAKY 480
XP_010702166.1_L.pan      VDGFYRYGRKLHYTADLVMALLWGCACGFKSFIPFFYFSFFLTHLIHRERRDEHRCKAKY 480
CCM18528.1_L.guy          VDGFYRYGRKLHYTADLVMALLWGCACGFKSFIPFFYFSFFLTHLIHRERRDEHRCKAKY 480
LSCM1_02556_L.mar         VDGFFRYGRKLHYTVDLVMAFLWGSACGFESFIPFFYFFFFLAHLVDRERRDENRCRAKY 476
KAG5469688.1_L.enr        VDGFYRYGRKLHYTVDLVMAFLWGSACGFDSFIPFFYFFFFLGHLVDRERRDEHRCKAKY 476
KAG5493492.1_L.Nam        VDGFYRYGRKLHYTADLVMAFLWGSACGFDSFIPFFYFFFFLGHLVDRERRDEHRCKAKY 476
KAG5494252.1_L.Gha        VDGFYRYGRKLHYTVDLVMAFLWGSACGFDSFIPFFYFFFFLGHLVDRERRDEHRCKAKY 476
KAG5468988.1_L.ori        VDGFYRYGRKLHYTVDLVMAFLWGSACGFDSFIPFFYFFFFLGHLVDRERRDEHRCKAKY 476
XP_003863897.1_L.don      VDGFYRYGRKLHYTVDLVMAFLWGSACGFESFIPFFYFFFFLGHLMDRERRDDHRCRAKY 476
XP_001468153.1_L.inf      VDGFYRYGRKLHYTVDLVMAFLWGSACGFESFIPFFYFFFFLGHLMDRERRDDHRCRAKY 476
XP_003878317.1_L.mex      VDGFYRYGRKLHYTVDLVMAFLWGSACGFESFIPFFYFFFFLCHLIDRERRDEHRCRAKY 476
                          ****:*********.*****:***.****.******** *** **:.*****::**:***

XP_001567842.1_L.bra      GKMWDEYVKLVPYKFIPYIY         500
XP_010702166.1_L.pan      GKMWDEYVKLVPYKFIPYIY         500
CCM18528.1_L.guy          GKMWDEYVKLVPYKFIPYIY         500
LSCM1_02556_L.mar         GKMWDDYVKLVPYKFIPYIY         496
KAG5469688.1_L.enr        GKMWDEYVKLVPYKFIPYIY         496
KAG5493492.1_L.Nam        GKMWDEYVKLVPYKFIPYIY         496
KAG5494252.1_L.Gha        GKMWDEYVKLVPYKFIPYIY         496
KAG5468988.1_L.ori        GKMWDEYVKLVPYKFIPYIY         496
XP_003863897.1_L.don      GKMWDEYVKLVPYKFIPYIY         496
XP_001468153.1_L.inf      GKMWDEYVKLVPYKFIPYIY         496
XP_003878317.1_L.mex      GKMWDEYVKLVPYRFIPYIY         496
                          *****:*******:******
```

Fig. S4 Blastp result of hypothetical protein LSCM1_02556 [*Leishmania martiniquensis*].
Description: hypothetical protein LSCM1_02556 [Leishmania martiniquensis]...
Molecule type: amino acid
Query Length: 496

Descriptions | Graphic Summary | Alignments | Taxonomy

Sequences producing significant alignments Download Select columns Show 100

select all *100 sequences selected* GenPept Graphics Distance tree of results Multiple alignment MSA Viewer

	Description	Scientific Name	Max Score	Total Score	Query Cover	E value	Per. Ident	Acc. Len	Accession
☑	hypothetical protein LSCM1_02556 [Leishmania martiniquensis]	Leishmania martiniquensis	1031	1031	100%	0.0	100.00%	496	KAG5469341.1
☑	hypothetical protein JIQ42_01860 [Leishmania sp. Namibia]	Leishmania sp. Namibia	865	865	100%	0.0	89.11%	496	KAG5493492.1
☑	hypothetical protein CUR178_01826 [Leishmania enriettii]	Leishmania enriettii	863	863	100%	0.0	89.11%	496	KAG5469688.1
☑	hypothetical protein GH5_02245 [Leishmania sp. Ghana 2012 LV757]	Leishmania sp. Ghana 2012 ...	853	853	100%	0.0	89.92%	496	KAG5494252.1
☑	hypothetical protein LSCM4_02382 [Leishmania orientalis]	Leishmania orientalis	852	852	100%	0.0	89.72%	496	KAG5468988.1
☑	putative sterol C-24 reductase [Leishmania mexicana MHOM/GT/2001/U1103]	Leishmania mexicana MHOM...	847	847	94%	0.0	86.97%	496	XP_003878317.1
☑	putative sterol C-24 reductase [Leishmania braziliensis MHOM/BR/75/M2904]	Leishmania braziliensis MHO...	841	841	100%	0.0	82.60%	500	XP_001567842.1
☑	sterol C-24 reductase, putative [Leishmania panamensis]	Leishmania panamensis	838	838	100%	0.0	82.60%	500	XP_010702166.1
☑	sterol C-24 reductase, putative [Leishmania guyanensis]	Leishmania guyanensis	837	837	100%	0.0	82.40%	500	CCM18528.1
☑	putative sterol C-24 reductase [Leishmania infantum JPCM5]	Leishmania infantum JPCM5	823	823	94%	0.0	86.97%	496	XP_001468153.1
☑	hypothetical protein JKF63_02082 [Porcisia hertigi]	Porcisia hertigi	813	813	100%	0.0	79.64%	496	KAG5495030.1
☑	putative sterol C-24 reductase [Leishmania major strain Friedlin]	Leishmania major strain Friedlin	791	791	88%	0.0	86.59%	441	XP_001685811.1
☑	putative mitochondrial sterol C-24 reductase [Leptomonas pyrrhocoris]	Leptomonas pyrrhocoris	778	778	94%	0.0	79.20%	503	XP_015655173.1

Fig. S5 Alignment of the protein sequences corresponding to the hypothetical protein LSCM1_01856 [*Leishmania martiniquensis*]. From top to bottom parasites listed are *L.* (*M.*) *martiniquensis*, *L.* sp. *Namibia* (*L.* (*M.*) *procaviensis* n. sp.), *L.* (*M.*) *enriettii*, *L.* (*M.*) *orientalis*, *L.* sp. *Ghana* (*L.* (*M.*) *chancei* n. sp.), *L.* (*V.*) *braziliensis*, *L.* (*V.*) *guyanensis*, *L.* (*V.*) *panamensis*, *L.* (*L.*) *mexicana*, *L.* (*L.*) *major*, (*L.*) *donovani*, *L.* (*L.*) *infantum*. Proteins sequences are from Blastp (https://blast.ncbi.nlm.nih.gov/Blast.cgi). Alignment was performed using Clustal Omega (https://www.ebi.ac.uk/jdispatcher/msa/clustalo). Note: The LSCM1_01856 sequence is similar to the ABC transporter-like protein/ABC transporter family protein sequences of other species.

```
CLUSTAL O(1.2.4) multiple sequence alignment


LSCM1_01856_L.mar         MVHLSTGICCTDAVLLAVSAANLPEFT-SSAPAMSPTTAVPADECGGMRQLLRFAYRYMT 59
KAG5494759.1_L.Nam        ---------------------------------MSSTTAAPAEETAGTRHLMRFAYRYMS 27
KAG5471028.1_L.enr        ---------------------------------MSSTTAAPAEDTAGTRHLMRFAYRYMS 27
KAG5470194.1_L.ori        ---------------------------------MLSTTAAPAEETAGTRHLMRFAYRYMS 27
KAG5495517.1_L.Gha        ---------------------------------MLSTTAAPAEETAGTRHLMRFAYRYMS 27
XP_001567581.1_L.bra      -----------------MSTAKLADSNSPSVPMVSSPTTAPAEEAVDTWQLVRFAYRYMV 43
CCM18281.1_L.guy          -----------------MSTAKLADLNSPSVPMVSSPTTAPAEEAVGTWQLVRFAYRYMV 43
XP_010701902.1_L.pan      -----------------MSTAKLADSNSPSVPMVSSPTTAPAEEAVGTWQLVQFAYRYMV 43
XP_003878054.1_L.mex      -----------------MSSAKYPESASPSARVVASPTTAPAGEAVGALHLLRFAYSYMT 43
XP_001685528.1_L.maj      -----------------MSSAKHPESGSPPAAVLSSPRTAPEGEAVGTLQLLRFAYSYMT 43
TPP47223.1_L.don          -----------------MSSAKHPESGSPPARVLSSPTTASESEAVGTLQLLRFAYSYMT 43
XP_001467897.1_L.inf      -----------------MSSAKHPESGSPPARVLSSPTTASESEAVGTLQLLRFAYSYMT 43
                                                           :    :.   :  .  :*::*** **

LSCM1_01856_L.mar         SQQRAVLISGLCMACATTLSISIPALAEEIISYGTETITKNSGGFPQASENTSATPPTGS 119
KAG5494759.1_L.Nam        SQQRMILVSGLFMACATLLSISIPALAEEIVSFGTETITKTLGGASHTAENSGVAPPTAA 87
KAG5471028.1_L.enr        SQQRMIVASGLFMACATLLSISIPALAEEIVSFGTETITKTLGGISYTAENSGVAPPTAA 87
KAG5470194.1_L.ori        SQQRIILVSGLFMACATLLSISIPALAEEIVSFGTETITKTLGGNSYTAENSGVAPPTAA 87
KAG5495517.1_L.Gha        SQQRMILVSGLFMACATLLSISIPALAEEIVSFGTETITKTLGGASHT-ANSGVAPPTAA 86
XP_001567581.1_L.bra      CEQRIILVSSFFMACATLLSISIPALAEEIVSYGMETITKTLNGVSHTVEDSRATSPAAA 103
CCM18281.1_L.guy          CEQRIILVSGFFMACATLLSISIPALAEEIVSYGMETITKTLNGVSHTVEDSRATSPAAA 103
XP_010701902.1_L.pan      CEQRIILVSGFFMACATLLSISIPALAEEIVSYGMETITKTLNGVSHTVEDSRATSPAAA 103
XP_003878054.1_L.mex      CQQRVILASGLFMTCATLLSISIPALAEEIVSYGMRTITKTIDSASPTAVSSSATSLEAA 103
XP_001685528.1_L.maj      CQQRVILVSGLFMACATLLSISIPALAEEIVSYGMRTITKTIDGVSHTAESSSATSLEAA 103
TPP47223.1_L.don          CQLRVILVSGLFMACATLLSISIPALAEEIVSYGMRATTKTIDGVSHTVESSSATSLEAA 103
XP_001467897.1_L.inf      CQLRVILVSGLFMACATLLSISIPALAEEIVSYGMRATTKTIDGVSHTVESSSATSLEAA 103
                          .: * :: *.: *:*** ************:*:* .: **. ..   :  .: .:   .:

LSCM1_01856_L.mar         ----GGWMPVDATRVGEDQLSFLGLGALSERMMKPLLPVVFAVLG---GGAVVGNNAEPL 172
KAG5494759.1_L.Nam        SASTGLGMPQDGAVVGADRLSILGLGPLSERMFKPVLPIFFALLS---GAAAVGNDAGPV 144
KAG5471028.1_L.enr        SASTGLGMPEDGTVVGADRLSILGLGPLSERMFKPVLPIFFALLG---GAASVGNDAGPV 144
KAG5470194.1_L.ori        SASTGLGMPEDGTVVGADRLSILGLGPLSERMFKPVLPIFFALLG---GAAAVGNDAGPV 144
KAG5495517.1_L.Gha        SASTGLGMPEDGTVVGADRLSILGLGPLSERMFKPVLPIFFALLG---GAAAVGNDAGHV 143
XP_001567581.1_L.bra      PGVYGLGMSAE----GADQFSFLGLGALSERICKPLLPIFFAVLRVVCGAVVLGTGAEAA 159
CCM18281.1_L.guy          PGVYGLGMSAE----GADQFSFLGLGALSERICKPLLPIFFAVLRVVCGAVVVGTGAEAA 159
XP_010701902.1_L.pan      PGVYGLGMSAE----GADQFSFLGLGALSERICKPLLPIFFAVLRVVCGAVVVGTGAEAA 159
XP_003878054.1_L.mex      AATSGLGMPAEGAGAGADQFSFLGLGPLSERIFKPLLPMFFTFLR---GAVAVGDGAEPA 160
XP_001685528.1_L.maj      TAAPGLGVPADGAGAGADRLFFLGLGPLSERIFKPLLPMLFAFLR---GAVV-GDGTESV 159
TPP47223.1_L.don          TAAPGLGMPADGAGAGGDRLFFLGLGPLSERIFKPLLPMLFAFLR---SAVVVRDDAEPA 160
XP_001467897.1_L.inf      TAAPGLGMPADGAGAGGDRLFFLGLGPLSERIFKPLLPMLFAFLR---SAVVVRDDAEPA 160
                              *  :  :    * *:: :**** ****: **:**:.*:.*    ...    .:

LSCM1_01856_L.mar         AWRSPYVEGILCRCLLMAAAVVCYHFTSLLAHLAAYYAGSGAQSALVRDSVKRILHTPHP 232
KAG5494759.1_L.Nam        VRLSPYVEGILCRCLLMAAAVTCYHFTSLLAHLAAYYAGSGAQSALIKDSVQRILHTPHP 204
KAG5471028.1_L.enr        VWLSPYVEGILCRCLLMAAAVTCYHFTSLLAHLAAYYAGSGAQSALMKDSVQRILHSPHP 204
KAG5470194.1_L.ori        VWLSPYVEGILCRCLLMAAAVTCYHFTSLLAHLAAYYAGSGAQSALMKDSVQRILHTSHP 204
KAG5495517.1_L.Gha        VWLSPYVEGILCRCLLMGAAVTCYHFTSLLAHLAAYYAGSGAQSALMKDSVQRILHTSHP 203
XP_001567581.1_L.bra      AWPSPYVEGILCRCLLMAMAIACYHATSLLAHLAAYYAGSGAQNALMKDSVHRILLTLHP 219
CCM18281.1_L.guy          AWPSPYVEGILCRCLLMAMAIACYHATSLLAHLAAYYAGSGAQNALMKDSVHRILLTLHP 219
XP_010701902.1_L.pan      AWPSPYVEGILCRCLLMAMAIACYHATSLLAHLAAYYAGSGAQNALMKDSVHRILLTLHP 219
XP_003878054.1_L.mex      VSLSPYAEGILCRCLLMSVAIICYHFTSLLAHLAAYYAGSGAQNALTKDSVQRILHTPHP 220
XP_001685528.1_L.maj      ASLSPYVEGVLCRCLLMAVAILCYHFTSLVAHLAAYYAGSGAQNALTKDSVQRILHTPRP 219
TPP47223.1_L.don          ASLSPYVEGILCRCLLMAVAIICYHFTSLLAHLAAYYAGSGAQNALTKDSVHRILHTPHP 220
XP_001467897.1_L.inf      ASLSPYVEGILCRCLLMAVAIICYHFTSLLAHLAAYYAGSGAQNALTKDSVRRILHTPHP 220
                          .  ***.**:*******. *: *** ***:*************.** :***:*** : :*

LSCM1_01856_L.mar         ERVAIVNSVKLAQLITTSGRALSDTMGELLTNVFSQVMYSVGFLAVMLFLSYRLTLTILV 292
KAG5494759.1_L.Nam        ERVAVVNAVKLAQLITTSGRALSDTMGELLTNVFSQLMYAAGFVAVMLLLSHRLTLTILV 264
KAG5471028.1_L.enr        ERVAVVNAVKLAQLITASGRALSDTMGELLTNVFSQLMYAAGFVAVMLLLSHRLTLTILV 264
KAG5470194.1_L.ori        ERVAVVNAVKLAQLITASGRALSDTMGELLTNVFSQLMYAAGFVAVMLLLSHRLTLTILV 264
KAG5495517.1_L.Gha        ERVAVVNAVKLAQLITASGRALSDTMGELLTNVFSQLMYAAGFVAVMLLLSHRLTLTILV 263
XP_001567581.1_L.bra      ERVTVVSAVKLAQLITASGRALSDTTGELLTNVLSQVMYTVGFFAVMLFLSYQLTLTILV 279
CCM18281.1_L.guy          ERVTVVSAVKLAQLITASGRALSDTTGELLTNVLSQVMYTAGFFAVMLFLSYQLTLTILV 279
XP_010701902.1_L.pan      ERVTVVSAVKLAQLITASGRALSDTTGELLTNVLSQVMYTVGFFAVMLFLSYQLTLTILV 279
XP_003878054.1_L.mex      ERVAVVNAVKLAQLITASGRALSETTGELLTNVLSQVMYIVGFFAVMLFLSYQLTLTILV 280
XP_001685528.1_L.maj      ERVAVVNAVKLAQLITASGRALSDTTGELLTNVLSQVMYIVGFFAVMLFLSYQLTLTILV 279
TPP47223.1_L.don          ERVAIVNAVKLAQLITASGRALSDTTGELLTNVLSQVMYIVGFFAVMLFLSYQLTLIILV 280
XP_001467897.1_L.inf      ERVAIVNAVKLAQLITASGRALSDTTGELLTNVLSQVMYIVGFFAVMLFLSYQLTLIILV 280
```

```
                           ***::*.:********:******:* *******:**:** .**.****:**::*** ***

LSCM1_01856_L.mar          GVVAVQCLFFFQGVSLHRKGSRVTAEEASMQAYITNILQRCQTVLVFGCSDFVLGRMANR 352
KAG5494759.1_L.Nam         GVVAVQCLFFFQGISLHRKGSRVTAGETSMQAYIANILQRSQTVLVFGCSDFVLGRMADR 324
KAG5471028.1_L.enr         GVVAVQCLFFFQGISLHRKGSRVTAEETSMQAYIANILQRSQTILVFGCSDFVLGRMAER 324
KAG5470194.1_L.ori         GVVAVQCLFFFQGISLHRKGSRVTAEETSMQAYIANILQRSQTVLVFGCSDFVLGRMADK 324
KAG5495517.1_L.Gha         GVVAVQCLFFFQGISLHRKGSRVTAEETSMQAYIANILQRSQTVLVFNCSDFVLGRMADK 323
XP_001567581.1_L.bra       GVVFVQCLFFFQGMSLHRQGCRVTAEEASVQSYIANVLQRSQTVLVFGCSDFVLSRMADR 339
CCM18281.1_L.guy           GVVLVQCLFFFQGMSLHRQGCCVTAEEASVQSYIANVLQRSQTVLVFGCSDFVLSRMADR 339
XP_010701902.1_L.pan       GVVLVQCLFFFQGMSLHRQGCCVTAEEASVQSYIANVLQRSQTVLVFGCSDFVLSRMADR 339
XP_003878054.1_L.mex       GVVGIQCLFFLQGISLHRQGSRLTAEEANVQAYIANILQRSQTVLVFGCSNFVLDRMEDR 340
XP_001685528.1_L.maj       GVVGIQCLFVLQGISLHRQGSRVTAEEASVQAYIANILQRSQTVLVFGCSEFVLDRMADR 339
TPP47223.1_L.don           GVVGIQCLFFFQGISLHRQGSRVTAEEASVQAYIANILQRSQTVLVFGCSEFVLDRMADR 340
XP_001467897.1_L.inf       GVVGIQCLFFFQGISLHRQGSRVTAEEASVQAYIANILQRSQTVLVFGCSEFVLDRMADR 340
                           *** :****.:**:****:*. :** *:.:*:**:*:***.**:***.**:***.** ::

LSCM1_01856_L.mar          SAEVRRLTNDLSLSIHGYAAVSSGLTRLILVVALGLSNYYQQRGQLNLRHTILYFACFQS 412
KAG5494759.1_L.Nam         SAEVRRLTNDLNLSIHGYAAVSSGLTRLILVVALGLSNYYQQKGQLNLRHTILYFACFQS 384
KAG5471028.1_L.enr         SAEVRRLTSDLNLSIHGYAAVSSGLTRLILVVALGLSNYYQQKGQLNFRHTILYFACFQS 384
KAG5470194.1_L.ori         SAEVRRLTNDLNLSIHGYAAVSSGLTRLILVVALGLSNYYQQKGQLNLRHTILYFACFQS 384
KAG5495517.1_L.Gha         SAEVRRLTNDLNLSIHGYAAVSSGLTRLILVVALGLSNYYQQKGQLNLRHTILYFACFQS 383
XP_001567581.1_L.bra       LAQVRRLTNSLNRSIHGYTAVSSGLTRLILVVALGLSNYYQQKGQLDIRHTILYFACFQS 399
CCM18281.1_L.guy           LAQVRRLTNSLNRSIHGYTAVSSGLTRLILVVALGLSNYYQQKGQLDMRHTILYFACFQS 399
XP_010701902.1_L.pan       LAQVRRLTNSLNRSIHGYTAVSSGLTRLILVVALGLSNYYQQKGQLDMRHTILYFACFQS 399
XP_003878054.1_L.mex       AAQLWRLTNGLNCSIHGYTAVSSALTRLILVVALGLSNYYQQKGQLDMRHTILYFACFQS 400
XP_001685528.1_L.maj       SAQLWRLTNGLNRSIHGYAAVSSGLTRLILVVALGLSNYYQQKGQLGMRHTILYFACFQS 399
TPP47223.1_L.don           SAQLWRLTNGLNRSIHGYAAVSSGLTRLILVVALGLSNYYQQKGQLGMRHTILYFACFQS 400
XP_001467897.1_L.inf       SAQLWRLTNGLNRSIHGYAAVSSGLTRLILVVALGLSNYYQQKGQLGMRHTILYFACFQS 400
                            *:: ***..*. *****:****.******************:***.:************

LSCM1_01856_L.mar          LVDTLVSLSSAVSQLRATLGRLKTLDAMLRWYSEPLAVAAGKDGTA------AAKLAVVD 466
KAG5494759.1_L.Nam         LVDTLVSLSSAVGQLRATLGRLKTLDAMLRWYSEPLAVATKEDGTA------ASTLGVVD 438
KAG5471028.1_L.enr         LVDTLVSLSSAVGQLRATLGRLKTLDAMLRWYSEPLAVATKEDGTA------ASTLGVVD 438
KAG5470194.1_L.ori         LVDTLVSLSSAVGQLRATLGRLKTLDAMLRWYSEPLAVAAKEDGTA------ASTLGVVD 438
KAG5495517.1_L.Gha         LVDTLVSLSSAVGQLRATLGRLKTLDAMLRWYSEPLAVAAKEDGTA------ASTLGVVD 437
XP_001567581.1_L.bra       FVNTLASLSSSVSQLRATLGRLRTLHSILRWYSEPLVVTTGEDGAA------ATPEAVVD 453
CCM18281.1_L.guy           FVNTLASLSSSVSQLRATLGRLRTLHSILRWYSEPLAVTTGEGGAA------ATPEAVVD 453
XP_010701902.1_L.pan       FVNTLASLSSSVSQLRATLGRLRTLHSILRWYSEPLAVTTGEGGAA------ATPEAVVD 453
XP_003878054.1_L.mex       FVSTLASLSSAVSELRATLGRLKTLDAMLRWYSEPLAVTA------GEGCAAASEAAVVD 454
XP_001685528.1_L.maj       LVNTLASLSSAVSQLRATLGRLKTLEAMLRWYSEPLADTA------GEGCTAASELAVVD 453
TPP47223.1_L.don           FVNTLASLSSAVSQLRATLGRLKTLDAMLRWYSEPLAGTA------GEGCTAALELAVVD 454
XP_001467897.1_L.inf       FVNTLASLSSAVSQLRATLGRLKTLDAMLRWYSEPLAGTAGEGGTAGEGCTVALELAVVD 460
                           :*.**.****:*.:********:**.::********. ::            *   .***

LSCM1_01856_L.mar          VQESSTAAVALEHVSFSYPAVPVFFREVG------NDAASLEEQLRTATGSQHNNGVSKV 520
KAG5494759.1_L.Nam         VQESATAAVALDHASFAYPTVPAFFREAG------DAAAALEEQLSPPTSSQHNNGVSQV 492
KAG5471028.1_L.enr         VQESATAAVALDNASFAYPAVPAFFREAG------DAAAALEEQLSSPTSSQHNNGVSQV 492
KAG5470194.1_L.ori         VQESATAAVALDHASFAYPAVPAFFREAS------DAAAALEEQLSSPTSSQHNNGVSQV 492
KAG5495517.1_L.Gha         VQESATAAVALDHASFAYPAVPAFFREAG------DAAAALEEQLSSPTSSQHNNGVSQV 491
XP_001567581.1_L.bra       VQESASAAVDLDHVSFSYPVVPAFFSEVNGATGAGNAVAALEKHLGSAMGSQHSNGVSQV 513
CCM18281.1_L.guy           VQESASAAVDLDHVSFSYPEVPAFFSEVNGATGAGNAVAALEKHLGSAMGSQHSNGVSQV 513
XP_010701902.1_L.pan       VQESASAAVDLDHVSFSYPEVPAFFSEVNGATGAGNAVAALEKHLGSAMGSQHSNGVSQV 513
XP_003878054.1_L.mex       VQESATADVALDHVSFSYPAVPAFLSEIGGAAGAGEAVASWEQELSTAMGSQHNNGVSQV 514
XP_001685528.1_L.maj       VQESATADVALDHVSFSYPAVPAFFSEVGGAAGAGDAVASWEQQLGTDMGSQHSNGVSEV 513
TPP47223.1_L.don           VQESPTAEVALDHVSFSYPAVPAFFSEVGGAAGAGDAVASWEQQLSTAMGSQHNNGVSQV 514
XP_001467897.1_L.inf       VQESPTAEVALDHVSFSYPAVPAFFSEVGGAAGAGDAVASWEQQLSTAMGSRHNNGVLQV 520
                           **** :* * *::.**:** **.*: * .      : .*: *:.*    .*:*.*** :*

LSCM1_01856_L.mar          SFTAAVGGITALYGPSGSGKSTCLRLLCGLVRPHEGIVRTQRRAVLLEQQHAIFMGSVAE 580
KAG5494759.1_L.Nam         SFTAAVGGITVLYGPSGSGKSTCLRLLCGLVRPHKGTVRTQRRAVLLEQQHAIFMGTVAE 552
KAG5471028.1_L.enr         SFTAAVGGITVLYGPSGSGKSTCLRLLCGLVRPHKGTVHTQRRAVLLEQQHAIFMGTVAE 552
KAG5470194.1_L.ori         SFTAAVGGITVLYGPSGSGKSTCLRLLCGLVRPHKGTVRTQRRAVLLEQQHAIFMGTVAE 552
KAG5495517.1_L.Gha         SFTAAVGGITVLYGPSGSGKSTCLRLLCGLVRPHKGTVRTQRRAVLLEQQHAIFMGTVAE 551
XP_001567581.1_L.bra       SFTAVVGGITVLYGPSGCGKSTCLRLLCGLVRPRTGTVHTQRRVVLLEQQHAIFIGTVAE 573
CCM18281.1_L.guy           SITAVVGGITVLYGPSGCGKSTCLRLLCGLVRPRTGTVHTQRRVVLLEQQHAIFMGTVAE 573
XP_010701902.1_L.pan       SITAVVGGITVLYGPSGCGKSTCLRLLCGLLRPRTGTVHTQRRVVLLEQQHAIFMGTVAE 573
XP_003878054.1_L.mex       SLTALVGGITVLYGPSGCGKSTCLRLLCGLVRPHTGTVRTQRRAVLLEQQHAIFIGTVAE 574
XP_001685528.1_L.maj       SLTALIGGITVLYGPSGCGKSTCLRLLCGLVRPHTGTVRTQRRALLLEQQHAIFIGTVAE 573
TPP47223.1_L.don           SLTALVGGITVLYGPSGCGKSTCLRLLCGLVRPHTGTVRTQRRAVLLEQQHAIFIGTVAE 574
XP_001467897.1_L.inf       SLTALVGGITVLYGPSGCGKSTCLRLLCGLVRPHTGTVRTQRRAVLLEQQHAIFIGTVAE 580
                           *:** :****.******.************:**: * *:****.:*********:*:***

LSCM1_01856_L.mar          NILLTDLSGFGRCTAGAGESKIAVPLSNGPHSPASAAAFAELQRRVTGAAVTSGCSGFLS 640
KAG5494759.1_L.Nam         NILLANLSDSVCCATNAGASKPTAPLSSVPLLLSPVAAFAELQRRVMDAAVKSGCTSFLS 612
KAG5471028.1_L.enr         NILLANLSDIVCCTTNAGASKPTAPLSSVHQLLSPVATFAELQRRVMDAAVKSGCTSFLS 612
KAG5470194.1_L.ori         NILLANLSDIVCCTTNAGASKPTAPLSSVPQLLSPVAAFAELQRRVMDAAVKSGCTSFLS 612
KAG5495517.1_L.Gha         NILLDNLSDIVCCTTNAGASKPTAPLSSVPQLLSPVAAFAELQRRVMDAAVKSGCTSFLS 611
XP_001567581.1_L.bra       NILLTNLSDLGTSMADAGASKPMLPLGHVPQSPTAAAAFAELHRRVTDAAAKSGCASFLK 633
CCM18281.1_L.guy           NILLTNLSDLGTSMADAGASKPMLPLGHVPQSPTAAAAFAELHRRVTDAAAKSGCASFLK 633
XP_010701902.1_L.pan       NILLTNLSDLGTSMADAGASKPMLPLGHVPQSPTAAAAFAELHRRVTDAAAKSGCASFLK 633
XP_003878054.1_L.mex       NILLTNLSSFGTSTTEVSALKPTMPSSGVPQSPTAAATFAELQRRVTDAAVKSGCANFLS 634
XP_001685528.1_L.maj       NILLTNLSSFGSSTAEASALKPTRPSSGALQLPTAAATFAEMQRRVTDAAVKSGCANFLS 633
TPP47223.1_L.don           NILLTSLSSFGSGTVKASALKPTMPSSGVPQLPTAAATFAELQRRVTDAAVKSGCADFLS 634
XP_001467897.1_L.inf       NILLTSLSSFGSGTAKASALKPTMPSSGVPQLPTAAATFAELQRRVTDAAVKSGCADFLS 640
                           **** .**.     . ..  *   * .      : .*:***::*** .**..***:.**.

LSCM1_01856_L.mar          NPFSTVIESVDHPQFSGGQLQRIALARVFARTDGYSLVLLDEPTTGLDQSAVEVLLRTIE 700
KAG5494759.1_L.Nam         NPFSTFIESIDHPQFSGGQLQRIALARVFARTDGYSLVLLDEPTTGLDQSAVEVLLETIK 672
```

```
KAG5471028.1_L.enr        NPFSTFIESVDHPQFSGGQLQRIALARVFARTDGYSLVLLDEPTTGLDQSAVEVLLETIK 672
KAG5470194.1_L.ori        NPFSTFIESVDHPQFSGGQLQRIALARVFARTDGYSLVLLDEPTTGLDQSAVEVLLETIK 672
KAG5495517.1_L.Gha        NPFSTFIESVDHPQFSGGQLQRIALARVFARTDGYSLVLLDEPTTGLDQSAVEVLLETIK 671
XP_001567581.1_L.bra      SPFSTFIESVDHPQFSGGQLQRIALARVFARTDGYSLVLLDEPTTGLDRNAVELLLESIR 693
CCM18281.1_L.guy          NPFSTFIESVDHPQFSGGQLQRIALARVFAQTDGYSLVLLDEPTTGLDRNAVELLLESIR 693
XP_010701902.1_L.pan      NPFSTFIESVDHPQFSGGQLQRIALARVFAQTDGYSLVLLDEPTTGLDRNAVELLLESIR 693
XP_003878054.1_L.mex      NPFSTFIESVDHPQFSGGQLQRIVLARMLARTDDYSLVLLDEPTTGLDRSAVEVLLEAIK 694
XP_001685528.1_L.maj      NPFSTFIESVDHPQFSGGQLQRIVLARMLARTDNHSLVLLDEPTTGLDRSAVEVLLEAIK 693
TPP47223.1_L.don          NPFSTFIESVDHPQFSGGQLQRIVLARMLARTDDYSLVLLDEPTTGLDRSAVEVLLETIK 694
XP_001467897.1_L.inf      NPFSTFIESVDHPQFSGGQLQRIVLARMLARTDDYSLVLLDEPTTGLDRSAVEVLLETIK 700
                          .****.***:*************.***::*:**.:*************:.***:**.:*.

LSCM1_01856_L.mar         ELRDVHHKTVLISTHDRRVAEVADKVVHLLASVAEAR   737
KAG5494759.1_L.Nam        ELRDIHHKTVLISTHDHRVAEVADKVIDLSTSVAEVR   709
KAG5471028.1_L.enr        ELRDIHHKTVLISTHDHRVAEVADKVIDLSTSVAEVR   709
KAG5470194.1_L.ori        ELRDIHHKTVLISTHDHRVAELADKVIDLSTSVAEVR   709
KAG5495517.1_L.Gha        ELRDIHHKTVLISTHDHRVAEVADKVIDLSTSVAEVR   708
XP_001567581.1_L.bra      ELRDTHH------------------------------   700
CCM18281.1_L.guy          ELRDTHHKTVLISTHDHRVAEVADKVIDMSTSAVETR   730
XP_010701902.1_L.pan      ELRDTHHKTVLISTHDHRVAEVADKVIDMSTSAVETR   730
XP_003878054.1_L.mex      ELRDTHHKTVLISTHDHRVAAVADKVINLSGSAAEVR   731
XP_001685528.1_L.maj      ELRDTHHKTILISTHDHRVAEVADKVIDLSASAAEAQ   730
TPP47223.1_L.don          ELRDTHHKTILISTHDRRVAEVADKVIDLSASAAEAR   731
XP_001467897.1_L.inf      ELRDTHHKTILISTHDRRVAEVADKVIDLSASAAEAR   737
                          **** **
```

Fig. S6 Blastp result of hypothetical protein LSCM1_01856 [*Leishmania martiniquensis*].
Description: hypothetical protein LSCM1_01856 [Leishmania martiniquensis]...
Molecule type: amino acid
Query Length: 737

Descriptions | Graphic Summary | Alignments | Taxonomy

Sequences producing significant alignments — Download — Select columns — Show 100

select all *100 sequences selected* — GenPept | Graphics | Distance tree of results | Multiple alignment | MSA Viewer

Description	Scientific Name	Max Score	Total Score	Query Cover	E value	Per. Ident	Acc. Len	Accession
hypothetical protein LSCM1_01856 [Leishmania martiniquensis]	Leishmania martiniquensis	1494	1494	100%	0.0	100.00%	737	KAG5470610.1
hypothetical protein LSCM4_02888 [Leishmania orientalis]	Leishmania orientalis	1156	1156	95%	0.0	80.54%	709	KAG5470194.1
hypothetical protein JIQ42_02374 [Leishmania sp. Namibia]	Leishmania sp. Namibia	1148	1148	95%	0.0	80.25%	709	KAG5494759.1
hypothetical protein GH5_03179 [Leishmania sp. Ghana 2012 LV757]	Leishmania sp. Ghana 201...	1146	1146	95%	0.0	80.25%	708	KAG5495517.1
hypothetical protein CUR178_02335 [Leishmania enriettii]	Leishmania enriettii	1145	1145	95%	0.0	79.69%	709	KAG5471028.1
ABC transporter family protein [Leishmania donovani]	Leishmania donovani	1047	1047	97%	0.0	74.22%	731	TPP47223.1
ABC transporter-like protein [Leishmania donovani]	Leishmania donovani	1043	1043	97%	0.0	73.75%	737	AYU81764.1
ABC transporter-like protein [Leishmania donovani]	Leishmania donovani	1040	1040	97%	0.0	73.15%	743	XP_003863633.1
ABC transporter-like protein [Leishmania infantum JPCM5]	Leishmania infantum JPCM5	1033	1033	97%	0.0	73.48%	737	XP_001467897.1
ABC transporter transmembrane region [Leishmania braziliensis]	Leishmania braziliensis	1032	1032	97%	0.0	71.64%	730	KAI5687610.1
ABC transporter-like protein [Leishmania braziliensis MHOM/BR/75/M2904]	Leishmania braziliensis M...	1030	1030	97%	0.0	71.51%	730	XP_001567581.1
Putative ABC transporter-like protein [Leishmania guyanensis]	Leishmania guyanensis	1030	1030	97%	0.0	71.64%	730	CCM18281.1
ABC transporter-like protein [Leishmania major strain Friedlin]	Leishmania major strain Fri...	1028	1028	97%	0.0	72.85%	730	XP_001685528.1
ABC transporter-like protein [Leishmania mexicana MHOM/GT/2001/U1103]	Leishmania mexicana MH...	1028	1028	97%	0.0	71.80%	731	XP_003878054.1
abc transporter-like protein [Leishmania tarentolae]	Leishmania tarentolae	1022	1022	94%	0.0	73.55%	739	GET91549.1
ABC transporter-like protein [Leishmania panamensis]	Leishmania panamensis	1002	1002	97%	0.0	71.51%	730	XP_010701902.1
